# Ultrasound‐Triggered Nanocomposite “Lever” Hydrogels with a Full Repair System Accelerates Diabetic Foot Ulcer Repair

**DOI:** 10.1002/advs.202500720

**Published:** 2025-05-08

**Authors:** Yuting Shen, Shaoyue Li, Xiaodong Hou, Jifeng Yu, Yuli Zhu, Chongke Zhao, Zhiyuan Niu, Xin Guan, Bing Xiong, Sirui Wang, Yifei Yang, Xiao Li, Liping Sun, Shengbo Wu, Bin Huang, Huixiong Xu, Haohao Yin

**Affiliations:** ^1^ Department of Ultrasound Institute of Ultrasound in Medicine and Engineering Zhongshan Hospital Fudan University Shanghai 200032 China; ^2^ Department of Medical Ultrasound Center of Minimally Invasive Treatment for Tumor Shanghai Tenth People's Hospital Ultrasound Education and Research Institute School of Medicine Tongji University Shanghai Engineering Research Center of Ultrasound Diagnosis and Treatment Shanghai 200072 China; ^3^ Department of Medical Ultrasound Henan Provincial People's Hospital School of Medicine Henan University Zhengzhou Henan Province 450003 China; ^4^ Department of Medical Ultrasound Affiliated Hospital of Nantong University Medical School of Nantong University Nantong Jiangsu Province 226001 China; ^5^ Ultrasound Department Zhejiang Hospital No. 1229 Gudun Road, Xihu District Hangzhou Zhejiang Province 310013 China

**Keywords:** anti‐inflammatory and antibacterial, diabetic foot ulcers, ergothioneine, nanocomposite “lever” hydrogels, reactive oxygen species regulation

## Abstract

Diabetic foot ulcers (DFUs) are a complex mixture of neuropathy, peripheral arterial disease, and infection, where excessive reactive oxygen species (ROS) exacerbates inflammation and impairs healing. Therefore, there is an urgent need to design a hydrogel dressing with a ‘lever’ function to balance ROS levels in the wound to achieve both antimicrobial and anti‐inflammatory effects on DFUs. In this study, we synthesised ROS‐responsive diselenide liposomes loaded with a pro‐skin healing factor (ergothioneine (ET)), thrombin, and a sonosensitizer (HMME) and constructed nanocomposite ‘lever’ hydrogels modulated by ultrasound (US). During early infection, sonodynamic therapy (SDT) under US generates bactericidal ROS, cleaving diselenide bonds to release ET and thrombin. Upon US cessation, thrombin/fibrinogen forms an in situ gel, while ET scavenges residual ROS and promotes M2 macrophage polarization in later stages. In addition, the potential immunomodulatory mechanisms of the nanocomposite ‘lever’ hydrogels were investigated via RNA sequencing. In conclusion, the novel nanocomposite ‘lever’ hydrogels effectively achieved a balance between ROS production and annihilation during different stages of wound repair while providing antibacterial and anti‐inflammatory properties to promote neovascularisation and improve diabetic peripheral neuropathy. In conclusion, by precisely controlling ROS levels across wound‐healing phases, this strategy offers a promising solution for refractory DFUs.

## Graphical abstract

Ultrasound (US)‐treated nanocomposite ‘lever’ hydrogels reprogrammed wound reactive oxygen species (ROS) levels and promoted wound healing in infected diabetic foot ulcers.

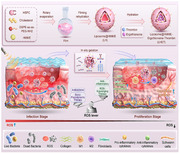

## 1 Introduction

Diabetes is the world's most common chronic disease, and diabetic foot ulcers (DFUs) are a common and serious complication of diabetes in the absence of therapeutic medications, occurring with a 34% probability.^[^ ^1^ ^]^ DFUs severely affect patients' quality of life, leading to lower limb amputations and death, thus creating a significant global health and economic burden.^[^ ^2^ ^]^ Current treatment modalities for DFUs include debridement, auto/‐/allografts and xenografts, cell‐based therapy and engineered skin grafts, weight‐bearing relief, hyperbaric oxygen therapy, anti‐infective treatments, and wound dressings.^[^ ^3^ ^]^ However, in clinical use, these methods have not produced satisfactory results for patients.^[^ ^4^ ^]^ The number of people with diabetes has sharply increased worldwide, and efficient treatments are presently lacking.^[^ ^5^ ^]^ Hence, it is important to discover novel treatments to promote wound healing in patients with DFUs.^[^ ^6^ ^]^ The dramatic increase in the number of diabetic patients worldwide and the lack of effective treatment strategies highlight the critical importance of the current discovery of new treatments for DFU wound healing.

Compared with normal wounds, persistent hyperglycemia not only causes bacteria at DFUs to proliferate widely, preventing the immune system from killing invading bacteria but also induces an excessive inflammatory response and disruption of the local immune microenvironment, culminating in the emergence of chronic nonhealing wounds.^[^ ^7^ ^]^ Therefore, addressing bacterial infection and excessive inflammation has become a major focus of current clinical DFU wound care.^[^ ^8^ ^]^ Sonodynamic therapy (SDT) is an excellent non‐antibiotic strategy that uses ultrasound (US) to activate acoustic sensitizer drugs, generating sufficient reactive oxygen species (ROS) to induce death in a wide range of drug‐resistant bacteria.^[^ ^9^ ^]^ Studies have shown that in the early stages of infection, ROS can cause intense oxidative stress and impaired energy production by disrupting the bacterial respiratory chain, ultimately killing bacteria.^[^ ^3^, ^10^ ^]^ However, prolonged and excessive ROS accumulation induces dysfunction of the immune response in the diabetic wound microenvironment, further triggering a severe inflammatory response and delaying wound healing.^[^ ^11^ ^]^ Therefore, the overproduction of ROS is a double‐edged sword, and we need both to pass ROS early in DFU wound therapy in order to be antimicrobial and to avoid the prolonged and excessive presence of ROS during DFU wound therapy.^[^ ^12^ ^]^ In addition, studies have shown that diabetic patients often have impaired antioxidant defenses.^[^ ^13^ ^]^ As a countermeasure, an antioxidant system with a “lever” function needs to be built up exogenously after the sterilizing effect of SDT in the early stages of infection to continuously scavenge the excess ROS generated by SDT and the high‐glucose microenvironment, thereby suppressing inflammation and promoting wound healing. Therefore, there is an urgent need to design a ROS‐modulated dressing system with a “lever” function based on the wound repair phase, which has both antimicrobial and anti‐inflammatory effects on DFU‐infected wounds.

Natural compounds have the potential to become exogenous antioxidant systems due to their high biosafety and wide range of pharmacological properties, thus functioning as “levers” to scavenge excess ROS.^[^ ^14^ ^]^ Ergothioneine (ET) is a sulfur‐containing amino acid derived from the ergot fungus, and its antioxidant effects are far superior to those of other common antioxidants, such as glutathione, vitamin C, and coenzyme Q10. The excellent antioxidant activity of ET is attributed to its ability not only to scavenge ROS directly and effectively chelate a variety of divalent metal cations but also to interact with the body's other natural antioxidant defense systems.^[^ ^15^ ^]^ Studies have shown that ET is 10 times more potent than glutathione and 4 times more potent than vitamin C in scavenging ROS at the same concentration.^[^ ^16^ ^]^ Importantly, as a natural compound of edible mushroom origin, ET has been recognized by the Food and Drug Administration (FDA) for its safety and stability. The consumption of ET within safe limits can improve the efficacy of clinical treatments for a wide range of conditions, including diabetes, organ fibrosis, neurological disorders, liver disorders, and pre‐eclampsia.^[^ ^15^, ^17^ ^]^ In addition, ET is preferentially exposed to cells and tissues damaged by oxidative stress in the body via the organic cation transporter protein OCTN1 in humans.^[^ ^18^ ^]^ Therefore, ET, due to its excellent ROS scavenging ability and biosafety, can play a “lever” function, making it very promising as a component of ROS‐modulated dressing system for scavenging excessive ROS generated by SDT in the early stages of infection and for eliminating inflammation induced by the persistent hyperglycemic microenvironment in subsequent wounds.

**Figure 1:**
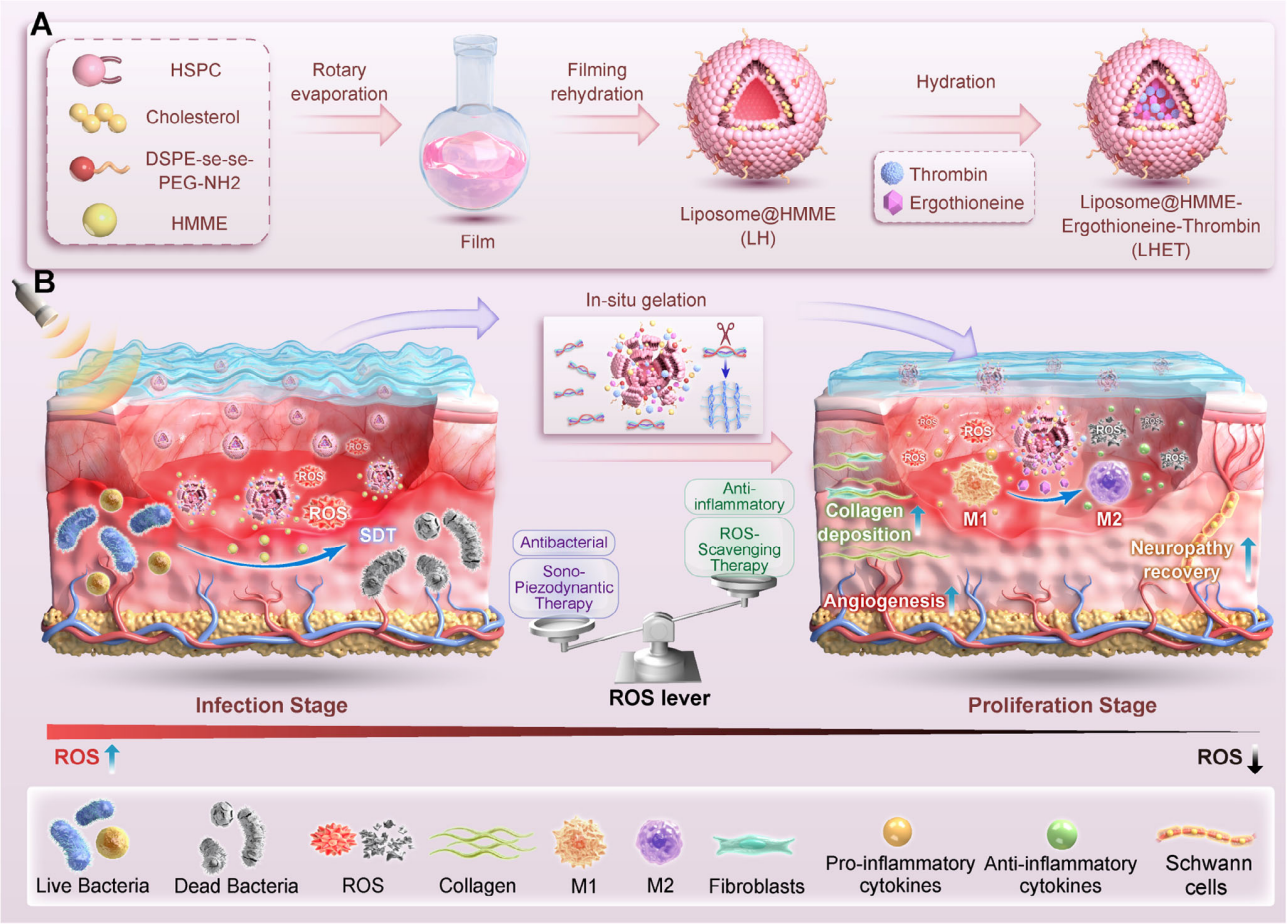
Schematic diagram of the DFU healing process programmed by the nanocomposite “lever” hydrogels. A) Flowchart for the synthesis of ROS‐responsive diselenide liposomes (LHET). B) US‐treated nanocomposite “lever” hydrogels reprogrammed wound ROS levels and promoted wound healing in infected DFUs. SDAT: sonodynamic antibacterial; ROS: reactive oxygen species.

Here, we propose a novel in situ nanocomposite hydrogel dressing with a “lever” function to programmatically regulate ROS levels in the wound microenvironment via the wound repair phase to achieve both antibacterial and anti‐inflammatory properties, ultimately facilitating DFU wound healing. Specifically, ET, thrombin, and an acoustic sensitizer (HMME) were first loaded into liposomes with a bis‐selenium bond (DSPE‐Se‐Se‐PEG‐NH2) via liposome extrusion to generate ROS‐responsive in situ nanoparticles (liposome@HMME‐ET‐thrombin nanoparticles, LHET NPs). In situ nanocomposite hydrogels were subsequently prepared on the basis of the enzymatic reaction of fibrinogen and thrombin.^[^ ^19^ ^]^ These novel in situ nanocomposite hydrogels can precisely fill irregularly shaped DFU wounds via the use of the ROS generated by SDT as a regulatory mechanism. At the initial stage of DFU wound infection, the rapid generation of ROS under US stimulation not only effectively eliminates bacteria from the wound surface but also destroys diselenide liposomes to release thrombin and ET. After the cessation of US, the in situ hydrogels (FLHET hydrogels) generated by the reaction of thrombin with fibrinogen ensure the continuous and slow release of ET during the wound healing phase. In vitro and in vivo studies have demonstrated that FLHET hydrogels with US stimulation have a comprehensive diabetic wound healing mechanism, including antimicrobial activity, induction of M2 macrophage polarisation, promotion of angiogenesis, and restoration of peripheral nerve function. In addition, this study preliminarily explored the potential immunomodulatory mechanisms of ultrasonically treated FLHET hydrogels in DFU wound healing via transcriptome sequencing. Overall, this study synthesized novel in situ nanocomposite hydrogels with a “lever” function on the basis of the rational regulation of ROS levels at different stages of wound repair, which provides a new therapeutic strategy for the treatment of refractory DFU‐infected wounds (**Figure** **1**)

## 2 Results and Discussion

### 2.1 Preparation and Characterization of LHET NPs

**Figure 2:**
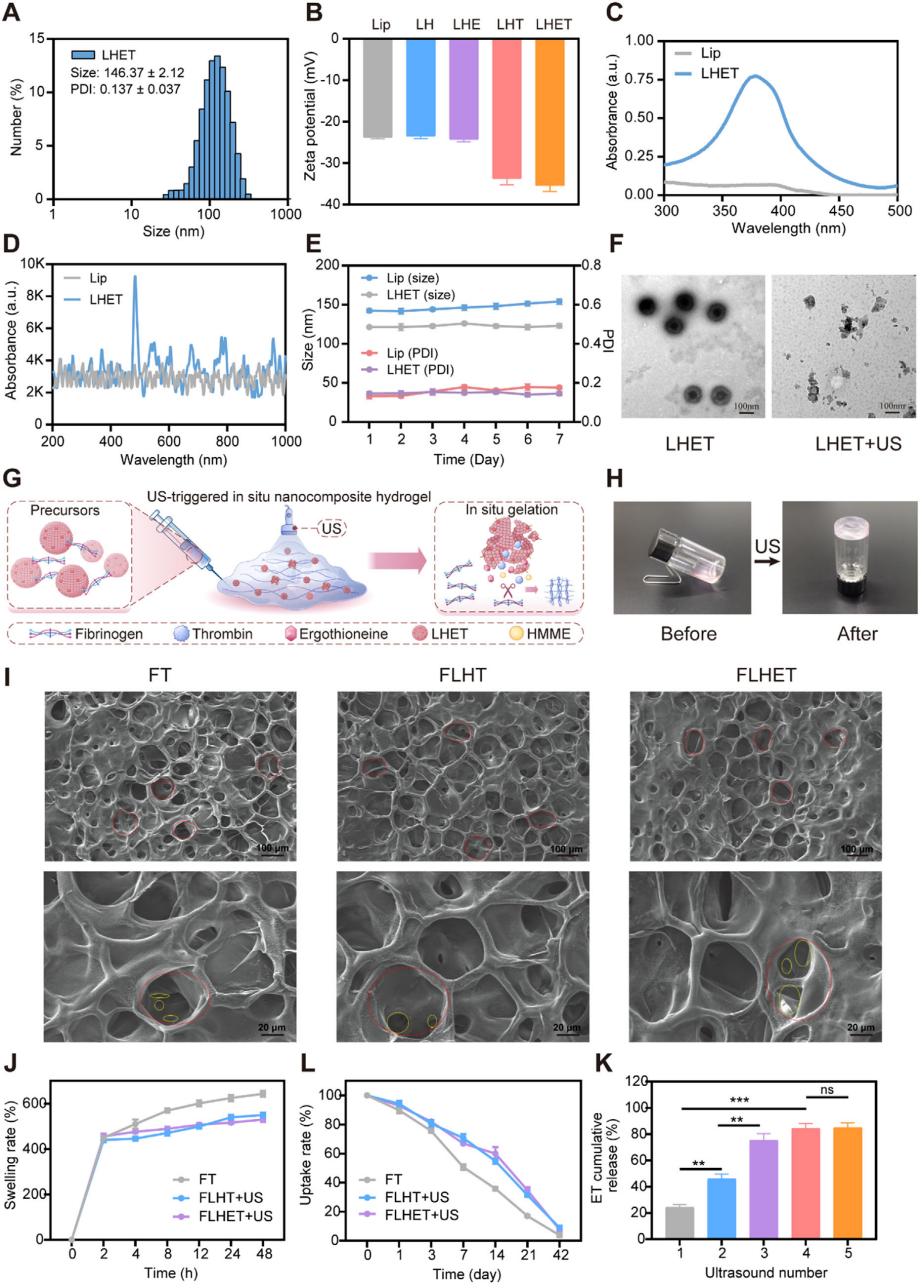
Characterisation of LHET NPs and the nanocomposite “lever” hydrogels. A) Size distribution of the LHET NPs. (B) Zeta potentials of Lip, LH, LHE, LHT and LHET. The values are presented as the means±SDs (*n* = 3). C,D) UV and Raman spectra of Lip and LHET NPs. E) Hydrodynamic sizes of Lips and LHET stored at 37 °C for 7 days. The values are presented as the means±SDs (*n* = 3). F) Representative TEM images of LHET NPs after sonication (scale bar: 100 nm). G) Schematic diagram of the in situ FLHET nanocomposite hydrogel preparation process. H) Images before and after gelation. I) SEM images of the FT, FLHT+US, and FLHET+US hydrogels. (Scale bar: 100 mm). J,L) The average swelling rates and uptake rates of the FT, FLHT+US, and FLHET+US hydrogels. K) The release of ET from the FLHET+US hydrogels at different US numbers. Values were indicated as mean±SD (*n* = 3). Significance between two groups was calculated using one‐way ANOVA and the ‐Tukey‐Kramer multiple comparisons test. ns = no significance, ^**^ *p* < 0.01, ^***^ *p* < 0.001.

Responsive liposomal NPs are excellent bioactive delivery vehicles extensively used for wound healing and in controlled delivery systems. The present study used a thin‐film hydration approach to prepare a ROS‐responsive diselenide liposome. To demonstrate that the liposomal diselenide bond can successfully respond to and break by ROS, we performed transmission electron microscopy (TEM). The Lip NPs showed a disrupted spherical structure after exposure to H_2_O_2_, suggesting that ROS production led to liposome rupture (Figure S1, Supporting Information). Dynamic light scattering (DLS) was conducted to confirm the size of the prepared LHET NPs. The NPs had a hydrodynamic size of 146.37±2.12 nm (PDI = 0.137±0.037) (**Figure** **2**A). The zeta potentials for liposome@HMME‐ET‐Thrombin (LHET), liposome (Lip), liposome@HMME (LH), liposome@HMME‐ET (LHE), and liposome@HMME‐Thrombin (LHT) were −35.42±1.43, −23.81±0.38, −23.56±0.48, −24.23±0.39, and −33.83±1.55 mV, respectively. LHET had a zeta potential close to that of LHT, which decreased relative to those of Lip, LH, and LHE; this finding indicated the effect of negatively charged thrombin protein on reducing the charge on liposomes (Figure 2B). UV and Raman spectra were used for qualitative analysis to assess whether LHET NPs were successfully prepared. The LHET solution presented unique UV peaks corresponding to HMME at 385 nm (Figure 2C). Furthermore, the Raman peak of ET was detected at 484 nm in the LHET solution (Figure 2D). Transmission electron microscopy (TEM) was conducted to confirm the morphology and size of the prepared ROS‐responsive LHET and Lip NPs. A uniform spherical shape was observed, and the diameter was ≈100 nm (Figure S2, Supporting Information). Additionally, to investigate whether LHET was stable, DLS was performed to measure its size at 37 °C for 7 days (Figure 2E). According to our observations, the LHET size mildly increased after 7 days. PDI was highly stable, indicating that LHET was highly stable (Figure 2E). Additionally, the Lip group was similar in size. To demonstrate that the rupture of LHET NPs results from the generation of ROS upon sonication, we observed TEM images of LHET and Lip NPs (with no HMME sensitizer as the control group) following 3 min of US treatment. TEM revealed that after US treatment, LHET NPs exhibited a broken spherical structure, whereas Lip NPs maintained their spherical structure; these findings indicated the role of US treatment in triggering ROS production, thereby causing cleavage of the double selenium bond and rupture of LHET NPs (Figure 2F).

### 2.2 Preparation and Characterization of the Nanocomposite “Lever” Hydrogels

In this study, US stimulation was used to fabricate nanocomposite “lever” hydrogels. Briefly, 100 mg mL^−1^ fibrinogen was subjected to 1 h of dissolution in a 37 °C water bath. Additionally, the BCA protein assay kit was used to quantify the thrombin concentration (100 UI mL^−1^) in the LHET NP solution. A syringe with an identical precursor volume was used to inject two FLHET hydrogel precursors. Following US treatment, a US sensitizer (HMME) was used for ROS (^1^O) generation. ROS induces the cleavage of the double selenium bond (DSPE‐Se‐Se‐PEG‐NH2) in the phospholipid bilayer of LHET NPs, leading to the breakage of LHET NPs and the release of thrombin and ET. The enzymatic reaction between thrombin and fibrinogen leads to the formation of FLHET NP hydrogels (Figure 2G). Prior to US treatment, the FLHET hydrogel precursor was liquid. This precursor underwent rapid liquid‐gel phase transition following 3 min of sonication (1 W cm^−2^, 1 MHz), indicating thrombin release from liposomes to induce in situ gelation (Figure 2H). To examine the efficiency of ROS production in LHET and LHT NPs within 3 min of sonication, we introduced 1,3‐diphenylisobenzofuran (DPBF) into the fibrinogen precursor to form an ROS (^1^O) sensitizer. As shown in Figure S3 (Supporting Information), the DPBF absorbance in the FLHET+US and FLHT+US hydrogels decreased dramatically in a time‐dependent manner, suggesting that the FLHET hydrogels have excellent ROS‐generating capacity in the presence of US treatment, suggesting the possibility of complete eradication of bacteria from DFU wounds (Figure S3, Supporting Information).

For the nanocomposite hydrogels, the pore structure included red‐labelled large pores and yellow‐labelled small pores. The mean sizes of the large pores in the FT, FLHT, and FLHET hydrogels were 149.63±5.14, 142.44±8.23, and 140.47±8.72 mm, respectively; these sizes were obtained on the basis of the internal hydrogel pore structure (Figure 2I). Notably, this porous structure with high interconnection and distribution levels contained numerous adhesion sites, thus creating a good microenvironment for the adhesion and migration of HUVECs. The swelling rates of the FLHET hydrogels were measured by weighing. As shown in Figure 2J, all the hydrogels exhibited rapid swelling behavior (more than 450%) during the first 2 h. All the hydrogels almost reached swelling equilibrium after 24 h, with the FT hydrogels showing the highest swelling rate (625%). Interestingly, the dissolution rates of the FHET hydrogels and FLHET hydrogels were similar, at 538% and 516%, respectively (Figure 2J). In addition, the dissolution rate of the FLHET hydrogels was 14.6% after 24 h of incubation in a wet environment, which did not change significantly compared with that of the initial hydrogels (Figure S4, Supporting Information). Therefore, the FLHET hydrogels did not cause side effects on the wounds because of excessive water absorption. The degradation of biomaterials provides space for tissue regeneration and, therefore plays a crucial role in the application of clinical wound aids.^[^ ^20^ ^]^ As shown in Figure 2L, the mass of the FT hydrogels decreased to 16.12% of the original mass after 21 days of PBS incubation, whereas the FLHET and FLHT hydrogels retained 34.57% and 32.24%, respectively (Figure 2L). These results indicate a slower degradation rate of in situ nanocomposite hydrogels than of FT hydrogels, which may be due to the formation of hydrogen bonds between the NH2‐residues of fibronectin in FLHT hydrogels and FLHET hydrogels and the OH‐groups of the DSPE‐Se‐Se‐PEG‐NH2 liposomes, which enhances the forces between the hydrogels and the liposomal molecules and ultimately reduces the nano‐degradation rate of the composite hydrogels. Additionally, the drug encapsulation rate is a critical factor for a drug delivery system. Fat‐soluble ET and HMME can be readily loaded onto the phospholipid bilayer of liposomes (LHET) at 85.6% and 89.7% encapsulation rates, respectively. In addition, the efficiency of ET release from FLHET hydrogels in the presence of US was assessed via Raman spectroscopy. To simulate the FLHET hydrogels at the beginning of DFU wound infection (within 24 h), we used US to treat the FLHET hydrogels in PBS at 6 h intervals for 3 min each time. As shown in Figure 2K, the level of ET release gradually increased with the number of US treatments, reaching a threshold (85.66%) after 4 US treatments, followed by a slow release of ET (Figure 2K). On the basis of these findings, the cumulative release rate of ET was correlated with the number of US sessions, with little change after reaching the threshold and only slow release.

### 2.3 The Biocompatibility of the Nanocomposite “Lever” Hydrogels

**Figure 3:**
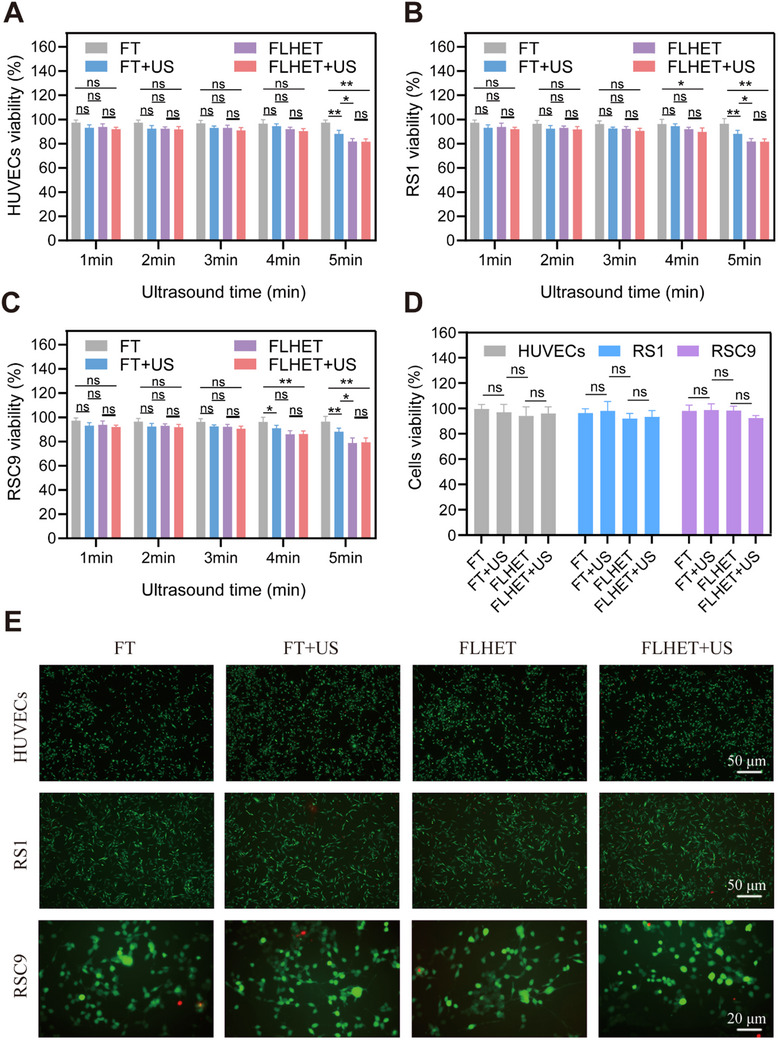
The biological safety evaluation of the nanocomposite “lever” hydrogels. A–C) HUVECs A), RS1 B), and RSC96 C) viability of the nanocomposite hydrogels under different US (1 W cm^−2^, 1 MHz) durations. The values are presented as the means±SDs (*n* = 5). D,E) Cells were treated with nanocomposite hydrogels accordingly for 24 h after CCK8. D) and live (green)/dead (red) cell staining E). Values were indicated as mean±SD (*n* = 3). Significance between the two groups was calculated using one‐way ANOVA and the Tukey–Kramer multiple comparisons test. ns = no significance, ^*^ *p* < 0.05, ^**^ *p* < 0.01.

Hydrogel biocompatibility is an important characteristic of “healing biomaterials,” which enable the maintenance of cell growth, differentiation, and survival. Furthermore, different sonication conditions may also lead to cell death. In the present study, we first assessed the activity of US alone on cells critical for wound healing by subjecting human umbilical cord mesenchymal stem cells (HUVECs), rat skin fibroblast‐like cells (RS1) and Schwann cell line (RSC96), Raw264.7, and keratinocyte migratory cells (Hacat) to US (1 W cm^−2^, 1 MHz) for 1–5 min, and subsequently assayed the different US treatment times on the toxicity of various cells. As shown in Figure S5A (Supporting Information), slight toxicity was present in Schwann cells and macrophages after US for more than 4 min, suggesting that the optimal duration of US treatment alone is 3 min (Figure S5A, Supporting Information). In addition, to assess the optimal biosafety concentrations of FT and FLHET, cells critical for wound healing were co‐cultured with different concentrations of FT and FLHET without US, respectively, for 24 h. The results showed that the FT hydrogels had excellent biocompatibility, however, some degree of toxicity was present in Schwann cells and macrophages when the FLHET concentration was more than 4 mg mL^−1^ (Figure S5B,C, Supporting Information). In order to evaluate the cell viability in various hydrogels after US (1 W cm^−2^, 1 MHz), we inoculated HUVECs, RS1, and RSC96 into different hydrogels, and then sonicated the four hydrogels accordingly for a total of 1–5 min. The results showed no statistically significant difference in cell viability between the two groups within 3 min of US. Nevertheless, after sonication for more than 4 min, some toxic effects were detected in the Schwann cell as determined by CCK‐8 assay (**Figure** **3**A–C). Finally, different hydrogels were inoculated with HUVECs, RS1, and RSC96 for a 24 h period with/without 3 min US treatment (1 W cm^−2^, 1 MHz). As shown in Figure 3D, the remaining level of ROS (produced after 3 min US treatment) following the redox reaction with the diselenide bond did not exhibit further toxic effects on cells (Figure 3D). The live/dead staining assay also confirmed that almost all cells in the FLHET nano‐in situ hydrogels group were alive (green) after 24 h of co‐incubation (Figure 3E). These findings showed that the nanocomposite “lever” hydrogels exhibited excellent biocompatibility and could serve as a matrix to support cell growth.

Biodegradation has an important effect on the use of biomaterials for adjusting the microenvironment and providing some space to achieve tissue regeneration. In the present study, we adopted a rat subcutaneous implantation model to assess hydrogel biodegradation. On postoperative days (PODs) 0, 7, 21, and 42, we collected the implanted hydrogels and neighboring tissues and assessed the remaining areas. The hydrogels gradually degraded. Typically, the implanted FT and FT+US hydrogels nearly disappeared on POD 21, whereas 42 days were needed for the complete degradation of the FLHET and FLHET+US hydrogels (Figure S6, Supporting Information). According to these findings, in situ nanocomposite “lever” hydrogels showed decreased degradation rates compared with those of pure fibrin hydrogels. This is probably because, in addition to the enzymatic reaction of thrombin and fibrinogen, hydrogen bonds were generated between the NH2‐residues in the fibrin hydrogels and the OH‐groups in the DSPE‐Se‐Se‐PEG‐NH2/HSPC liposomes in the FLHT/FLHET nanocomposite hydrogels, thus increasing the intermolecular forces of the hydrogels with liposome molecules while reducing the degradation rate of the nanocomposite hydrogels. The longer sustained degradation of the nanocomposite hydrogels provides adequate time and opportunity for subsequently improving diabetic foot neuropathy. Body weight did not significantly change in any of the hydrogel groups following treatment (Figure S7, Supporting Information). Histological analysis of the major organs obtained from chronic DFU rats after 21 days of treatment revealed no histopathological abnormalities (Figure S8, Supporting Information). In addition, peripheral blood was collected from rats in all groups on day 21, followed by blood biochemistry and hematology tests. As shown in Figure 6, the biochemical and hematological indices were normal in all groups, which indicated that the nanocomposite hydrogel had excellent biocompatibility (Figure S9, Supporting Information). Finally, to assess the targeting effect of ET, chronic DFU was collected from the vital organs and corresponding skin tissues of rats after 7 days of FLHET+US treatment, followed by the detection of ET content by Raman spectroscopy, which demonstrated that ET was mainly concentrated in the DFU wound and had an excellent targeting effect (Figure S10, Supporting Information). Consequently, in situ nanocomposite “lever” hydrogels used locally in vivo showed no significant toxic effects.

### 2.4 Antioxidant and Antimicrobial Properties of the Nanocomposite “Lever” Hydrogels

**Figure 4:**
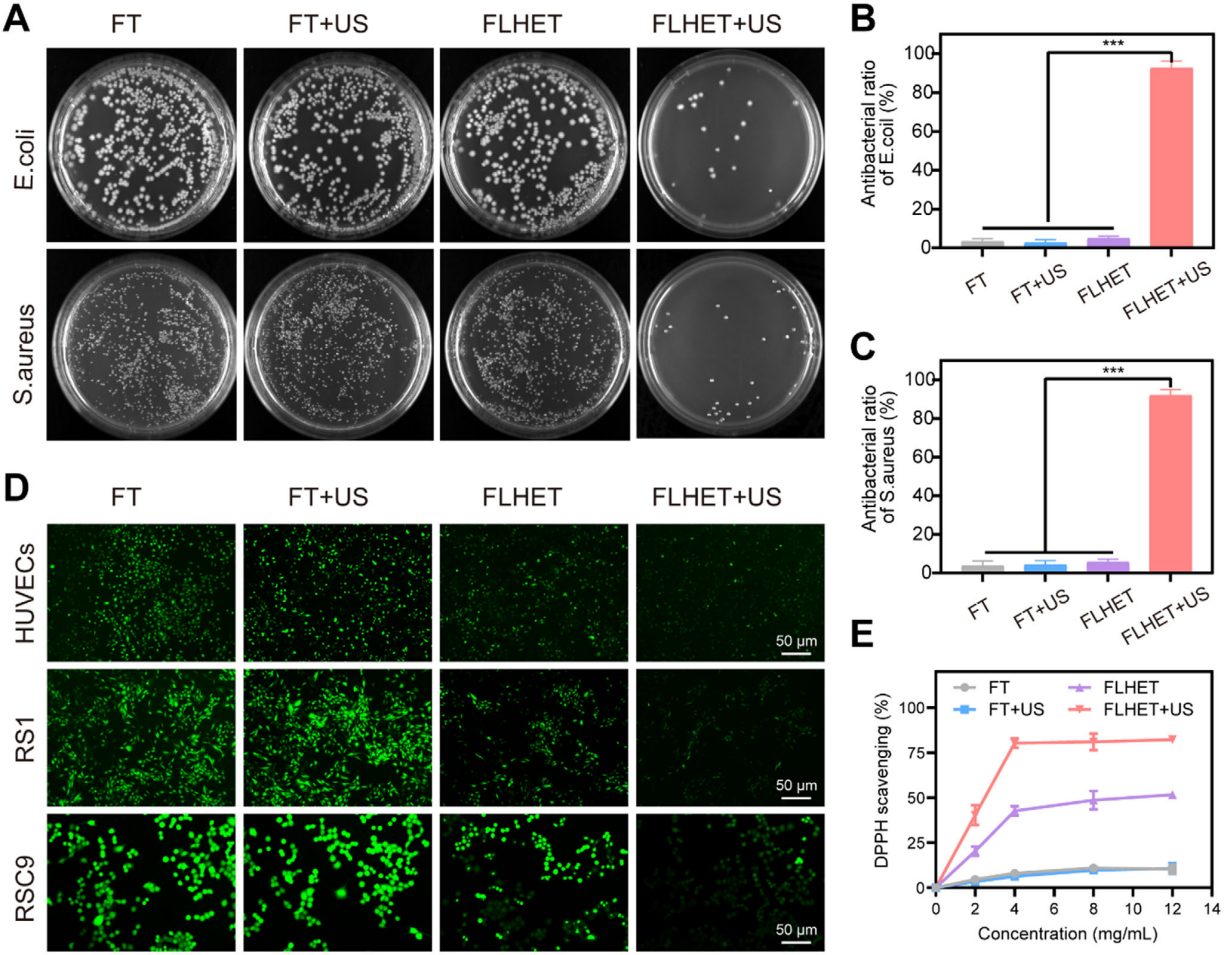
Evaluation of the antioxidant and antimicrobial capacity of the nanocomposite “lever” hydrogels. A) Images of *S. aureus* and *E. coli* colonies on Luria‐Bertani (LB) agar plates after treatment with FT hydrogels, FT+US hydrogels, FLHET hydrogels, and FLHET+US hydrogels. B,C) Bacterial survival of *S. aureus* and *E. coli* after different treatments. The values are presented as the means±SDs (*n* = 5). D) Intracellular ROS levels after treatment with different hydrogels. E) Clearance of DPPH after hydrogel treatment with different concentrations. Values were indicated as mean±SD (*n* = 5). Significance between the two groups was calculated using one‐way ANOVA and the Tukey‐Kramer multiple comparisons test. ns = no significance, ^*^ *p* < 0.05, ^**^p < 0.01.

The persistent hyperglycaemic microenvironment of the DFUs wound microenvironment is susceptible to bacterial infection.^[^ ^21^ ^]^ Effective antimicrobial therapy in the early stages of infection is essential to prevent the progression of refractory DFUs wounds.^[^ ^22^ ^]^ Given the high ROS production capacity of the FLHET hydrogels under US, the bacterial inhibitory capacity of the FLHET hydrogels was evaluated in gram‐positive Staphylococcus aureus (*S. aureus*) and Escherichia coli (*E. coli*). As shown by the results of bacterial plate culture, the FLHET hydrogels effectively inhibited the growth of bacteria after US, and the inhibition rate of both *S. aureus* and *E. coli* reached more than 90%, which demonstrated the excellent antimicrobial ability of the FLHET hydrogels (**Figure** **4**A–C). However, the accumulation of excess ROS in the DFU wound environment leads to chronic oxidative stress and inflammation, and the timely removal of large amounts of ROS generated by SDT is particularly important for DFU wounds in the proliferative phase.^[^ ^23^ ^]^ First, the antioxidant properties of the FLHET hydrogels in cells were investigated via an ROS fluorescent probe. Pretreatment of HUVECs and the RS1 and RSC9 cell lines with the classical inducer of oxidative stress, tert‐butyl hydroperoxide (TBHP), for 24 h resulted in high intracellular production of ROS. Cells were subsequently inoculated into four groups of hydrogels and incubated for 24 h, where the FT+US and FLHET+US groups were correspondingly sonicated (1 W cm^−2^, 1 MHz) for 3 min. As shown in 4D, the overproduced ROS were cleared by ET in the FLHET hydrogel group and the FLHET+US hydrogel group, whereas the ROS were cleared most completely in the FLHET+US hydrogel group, which may be attributed to the fact that the SDT‐mediated production of ROS disrupted the liposomes, which in turn released more ET (Figure 4D). In addition, 1,1‐Diphenyl‐2‐trinitrophenylhydrazine (1,1‐Diphenyl‐2‐picrylhydrazyl, DPPH) free radical scavenging assay could be used to further examine the ability of FLHET hydrogels to scavenge ROS in vitro. Although Figure S3 (Supporting Information) data have shown that FLHET+US generates ROS after US (3min, 1 W) treatment, however, ET released from FLHET+US hydrogel could continuously and slowly scavenge a large amount of ROS. Therefore, DPPH was examined in vitro after 12 h, and the results showed that the DPPH scavenging efficiency was higher in the FLHET+US hydrogel group than in the other groups. The antioxidant capacity of FLHET+US hydrogel was above 80% when the concentration of FLHET+US hydrogel was as high as 4 mg mL^−1^, indicating that the ET released from the hydrogel had excellent free radical scavenging properties (Figure 4E). In conclusion, the above results demonstrated that the ultrasonically treated FLHET hydrogels had excellent antioxidant and antimicrobial capabilities and could be used as promising dressings for DFUs wound therapy.

### 2.5 In Vitro Healing‐Promoting Properties of the Nanocomposite “Lever” Hydrogels

**Figure 5:**
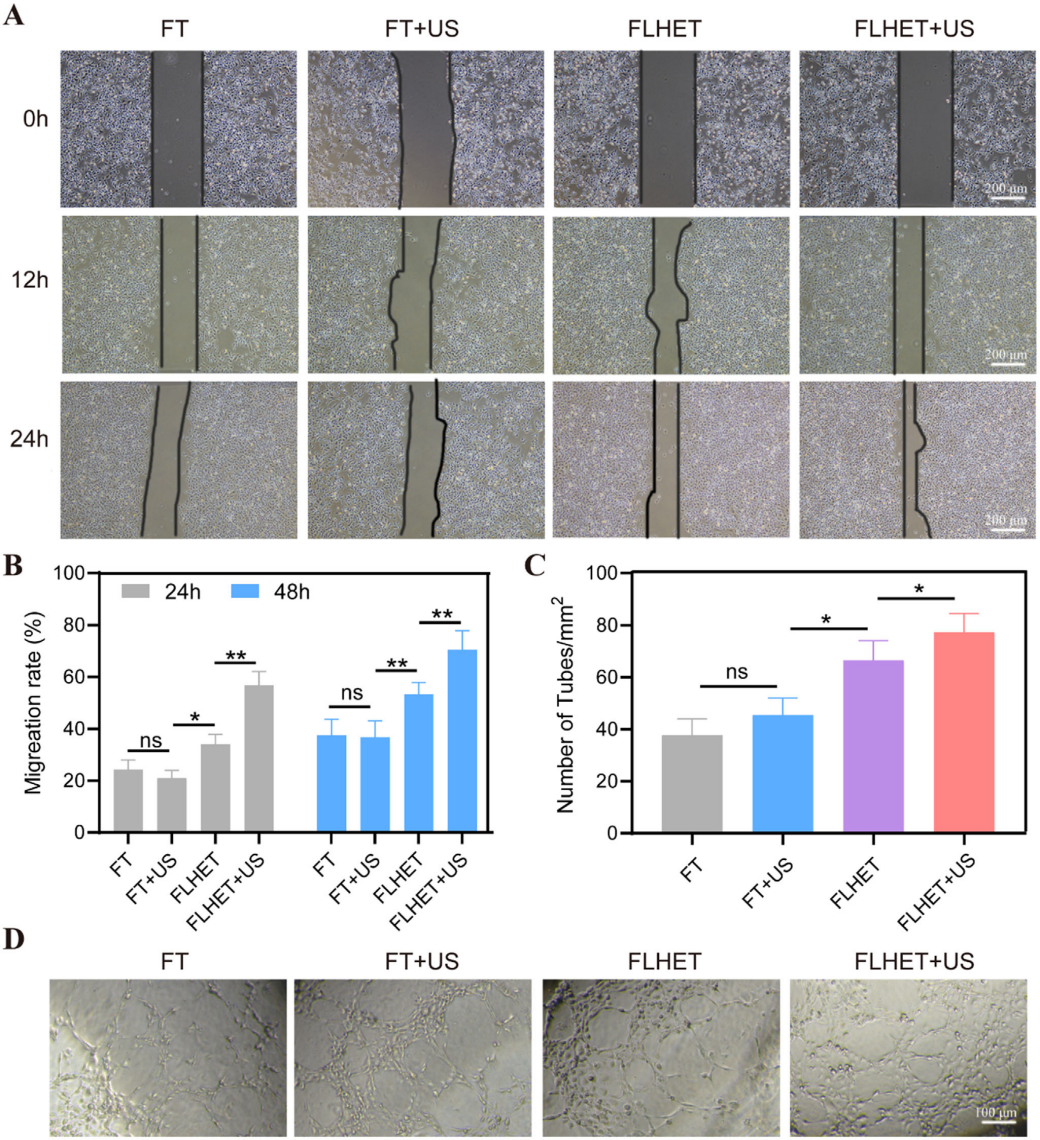
The healing capacities of the nanocomposite “lever” hydrogels in vitro. A,B) Optical images A) and quantitative results B) of the HUVECs scratch assay at 0, 12, and 24 h in different treatment groups. The values are presented as the means±SDs (*n* = 4). C) Optical images of HUVECs that formed tubes after different treatments. D) Quantitative results of tube formation in HUVECs after different treatments. Values were indicated as mean±SD (*n* = 4). Significance between two groups was calculated using one‐way ANOVA and the Tukey–Kramer multiple comparisons test. ns = no significance, ^*^ *p* < 0.05, ^**^ *p* < 0.01.

HUVECs were incubated with US (3 min, 1 W cm^−2^)‐treated FLHET hydrogels to examine their in vitro healing ability. As shown in **Figure** **5**A, a scratch test was conducted to assess the in vitro healing capacity of the FLHET hydrogels. The FLHET and FLHET+US hydrogel groups presented gradually increasing migration rates with time, with 45% and 80% migration rates at 24 h, respectively. Compared with the FLHET group, the FLHET+US hydrogel group significantly promoted the migration and cell‐free gap closure of HUVECs (Figure 5B). We also performed an endothelial tube formation assay to determine in vitro angiogenesis in FLHET hydrogels. Compared with the FLHET hydrogel group, the FLHET+US hydrogel group formed the densest network of tubes after 6 h of incubation (Figure 5C,D). The results showed that the FLHET+US hydrogels exhibited favorable cytocompatibility, which substantially enhanced the growth and in vitro migration of HUVECs.

### 2.6 Nanocomposite “Lever” Hydrogels Accelerated Healthy and Diabetic Wound Healing In Vivo

**Figure 6:**
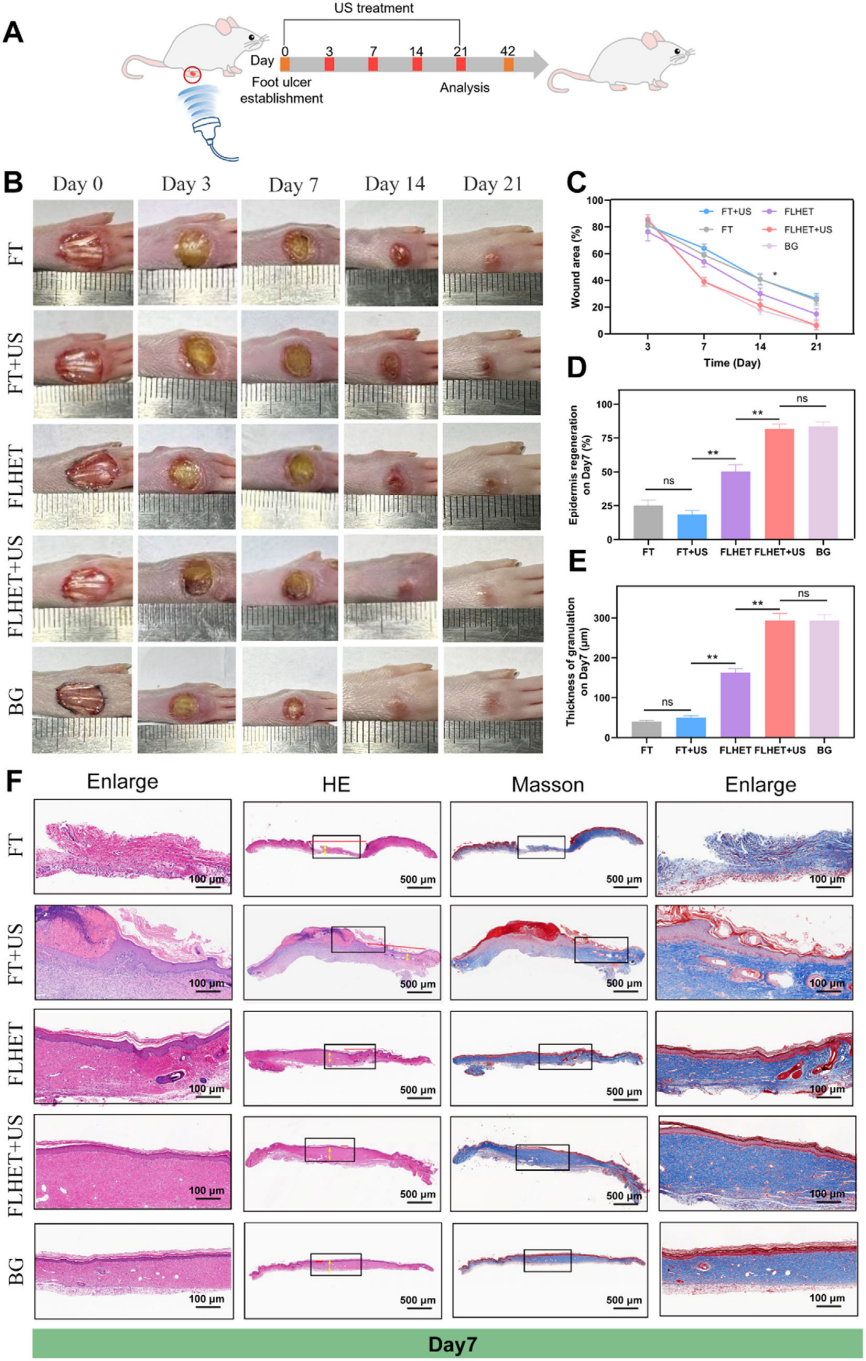
In vivo wound healing of the nanocomposite “lever” hydrogels. A) Schematic representation of the treatment strategy for chronic DFU model infection in different treatment groups. B) Optical photos of wound closure within 21 days after different treatments. C) Statistical analysis of wound healing on the basis of the wound areas. The values are presented as the means±SDs (*n* = 4). D) Quantitative statistics of epidermal regeneration rates after different treatments. The values are presented as the means±SDs (*n* = 4). E) Quantitative statistics of granulation tissue regeneration thickness after different treatments. The values are presented as the means±SDs (*n* = 4). F) H&E staining and Masson's trichrome staining of the wounded tissue after different treatments. The red line arrows represent the length of the wound. The yellow arrows represent the thickness of the granulation tissue. Values were indicated as mean±SD (*n* = 4). Significance between the two groups was calculated using one‐way ANOVA and the Tukey–Kramer multiple comparisons test. ns = no significance, ^*^ *p* < 0.05, ^**^ *p* < 0.01.

Following in vitro assays, we constructed a diabetic rat model induced by streptozotocin (STZ) with full‐thickness skin wounds to evaluate the efficacy of the FLHET hydrogel in vivo. In addition, in order to further fully evaluate the therapeutic efficacy of FLHET+US hydrogels, the FDA approved for the treatment of DFUs hydrogels Becaplermin gels (BG) can be used as a positive control group, providing the possibility of FLHET+US Hydrogel being used in clinical treatment. The schedule of treatment is summarized in **Figure** **6**A. Diabetic rat foot ulcer wounds in the FT, FT+US, FLHET, FLHET+US, and BG groups were simultaneously managed. US exposure/unexposure was then conducted at 1 W cm^−2^ power with a 50% duty cycle and a 1 MHz frequency for 3 min on days 0, 3, 7, 14, and 21. Digital photographs of the wounds showed that similar to the BG group, FLHET+US hydrogel achieved wound healing rates of ≈76% on day 14 and ≈100% on day 21. (Figure 6B,C). Subsequently, histological analyses were performed via hematoxylin‐eosin (H&E) staining and Masson's trichrome staining to assess the quality of wound healing on day 7. Compared with the other groups, the FLHET+US hydrogel and BG groups presented faster wound contraction, a more intact epidermal layer, and more orderly and thicker granulation tissue. The results of Masson staining revealed that the FLHET+US hydrogel and BG groups presented a darker blue color than the other groups did, which proved that more collagen was deposited in the skin tissue and that the collagen fibers were denser and more evenly arranged (Figure 6D–F). In contrast, the FT hydrogel group presented only a small number of loose and disorganized collagen fibers, and further quantitative statistics are shown in Figure S11 (Supporting Information). In conclusion, these results indicated that the FLHET+US hydrogel effectively promoted DFU wound healing.

The dynamic course of ROS levels on the surface of DFU wounds from the infection phase to the proliferation phase was monitored by tissue immunofluorescence. Compared with the other groups, during the wound infection stage on day 1, the FLHET+US hydrogel group presented significant fluorescence enhancement, indicating that a large amount of ROS could be generated by SDT (Figure S12, Supporting Information). Moreover, the fluorescence intensity of the FLHET+US hydrogel and BG groups was significantly lower on day 4 than that of the FT hydrogel group and FT+US hydrogel group, suggesting that ET excellently and consistently scavenges ROS (Figure S13, Supporting Information). In addition, the antioxidant capacity of FLHET+US hydrogel in vivo could be further assessed by measuring the extracellular matrix of DFU wounds. After 14 days of hydrogel corresponding treatment in each group, we assessed the expression of Col‐1 in DFU wounds in each group, and the results showed that the highest expression of (Collagen I) Col‐1 was found in the FLHET+US hydrogel and BG groups compared to the other groups (Figure S14, Supporting Information). Therefore, the FLHET+US hydrogel group exhibited excellent SDT‐induced ROS generation during the DFU wound infection stage, which resulted in bacterial clearance, whereas the released ET removed excess ROS from the tissues during the proliferation stage, which ultimately led to the dynamic regulation of local ROS in the wound.

Additionally, α‐SMA (red) with CD31 (green) double staining was performed to analyze angiogenesis. CD31 is a marker for newly formed blood vessels at the wound site, whereas α‐SMA is a marker for mature blood vessels. The CD31 and α‐SMA levels in the FLHET+US hydrogel and BG groups were significantly greater at 7 days post‐operation, suggesting that the “lever” hydrogels enhanced angiogenesis and accelerated wound healing at an early stage (Figure S15, Supporting Information). Given that ET ameliorates chemotherapeutic drug‐induced peripheral neuropathy in rats, we investigated the potential of “lever” hydrogels in ameliorating peripheral nerve dysfunction in rats with diabetic foot ulcers. Intraepidermal nerve fibers (IENFs) are directly associated with the functional innervation of the skin, and their reduction is one of the main histological characteristics of human diabetic polyneuropathy. The results of the immunohistofluorescence assay revealed that IENF expression was significantly increased in the skin of the footpads in the FLHET+US hydrogels and BG groups compared with that in the control group (Figure S16A,B, Supporting Information). In addition, compared with the other groups, the FLHET+US hydrogel and BG groups presented significant reductions in thermal hyperalgesia and partial restoration of mechanical sensitivity at different postoperative time points (Figure S16C,D, Supporting Information). These results indicate that the nanocomposite “lever” hydrogels are potential agents for treating chronic DFUs.

### 2.7 Nanocomposite “Lever” Hydrogels Decreased the Expression of Genes and Pathways Associated with the Inflammatory Response

**Figure 7:**
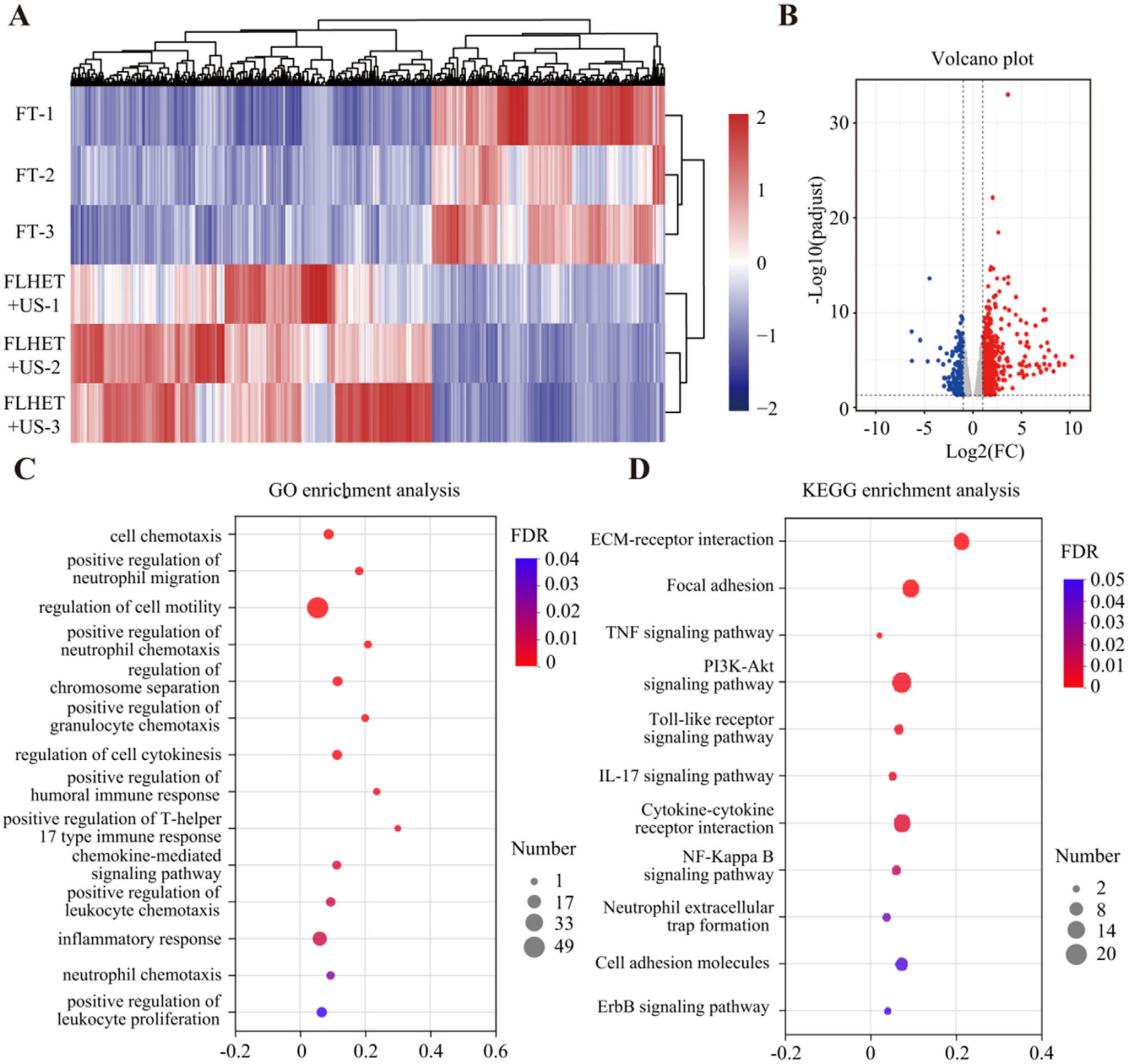
Global assessments of the effects of “lever” hydrogels on gene expression in DFU wounds. A) Volcano plot of differentially expressed genes (DEGs) in the RNA from the wound tissue after different treatments. B) Venn diagram of the gene characteristics of wound tissue after different treatments. C) GO enrichment analysis of the DEGs in the “lever” hydrogel group. D) KEGG enrichment analysis of DEGs in the “lever” hydrogel group.

To investigate the molecular mechanisms underlying the pro‐healing capacity of the nanocomposite “lever” hydrogels, foot ulcer tissues were obtained from diabetic rats and examined via RNA sequencing (RNA‐seq). A total of 869 upregulated differentially expressed genes (DEGs) (false discovery rate (FDR) < 0.05, fold change (FC) ≥2 or ≤ 0.5) and 521 downregulated DEGs were obtained from the volcano plot. These findings demonstrated that US‐treated FLHET hydrogels altered gene expression patterns in chronic DFUs (**Figure** **7**A,B). These DEGs were subsequently subjected to functional annotation. Gene Ontology (GO) and Kyoto Encyclopedia of Genes and Genomes (KEGG) enrichment analyses revealed that FLHET hydrogels decreased the expression of genes related mostly to the immune response, inflammation, and chemokine‐regulated pathways on POD 7 (Figure 7C,D). Persistent and impaired inflammation impedes tissue healing. The FLHET+US hydrogels exerted anti‐inflammatory effects, thus facilitating diabetic wound healing. Gene output was compared via the early‐stage inflammation signature. The results revealed that nearly all inflammation‐related genes were downregulated in the FLHET+US hydrogel group at POD 7, whereas those encoding regeneration‐related proteins were upregulated (Figure S17, Supporting Information).

### 2.8 Nanocomposite “Lever” Hydrogels Mitigated the Inflammatory Response by Modulating the PI3K/AKT/NF‐κB Pathway

Uncontrolled macrophage activation in chronic diabetic wounds results in the production of excessive amounts of cytokines and amplifies the inflammatory response, thus leading to chronic inflammation. Excessive inflammation is the major cause of chronic diabetic wounds because the ongoing inflammatory phase rarely transitions to the proliferative phase. Here, RAW 264.7 mouse macrophages were incubated with US‐treated FLHET hydrogels to examine the in vitro anti‐inflammatory capabilities of the hydrogels. As shown in Figure S18 (Supporting Information), LPS‐stimulated RAW 264.7 cells generated numerous inflammatory factors; however, neither the FT+US nor the FLHET hydrogel group presented a decrease in their intracellular contents. In contrast, in the FLHET+US hydrogel group, the levels of these inflammatory factors were significantly reduced in the LPS‐treated RAW 264.7 cells (Figure S18, Supporting Information). Additionally, immunohistochemistry and its quantitative results indicated that FLHET+US hydrogels significantly reduced the levels of inflammatory factors such as IL‐6, TNF‐α, and IL‐1β in the wound tissue (Figure S19, Supporting Information). These results demonstrate the anti‐inflammatory effect of FLHET hydrogels under US.

**Figure 8:**
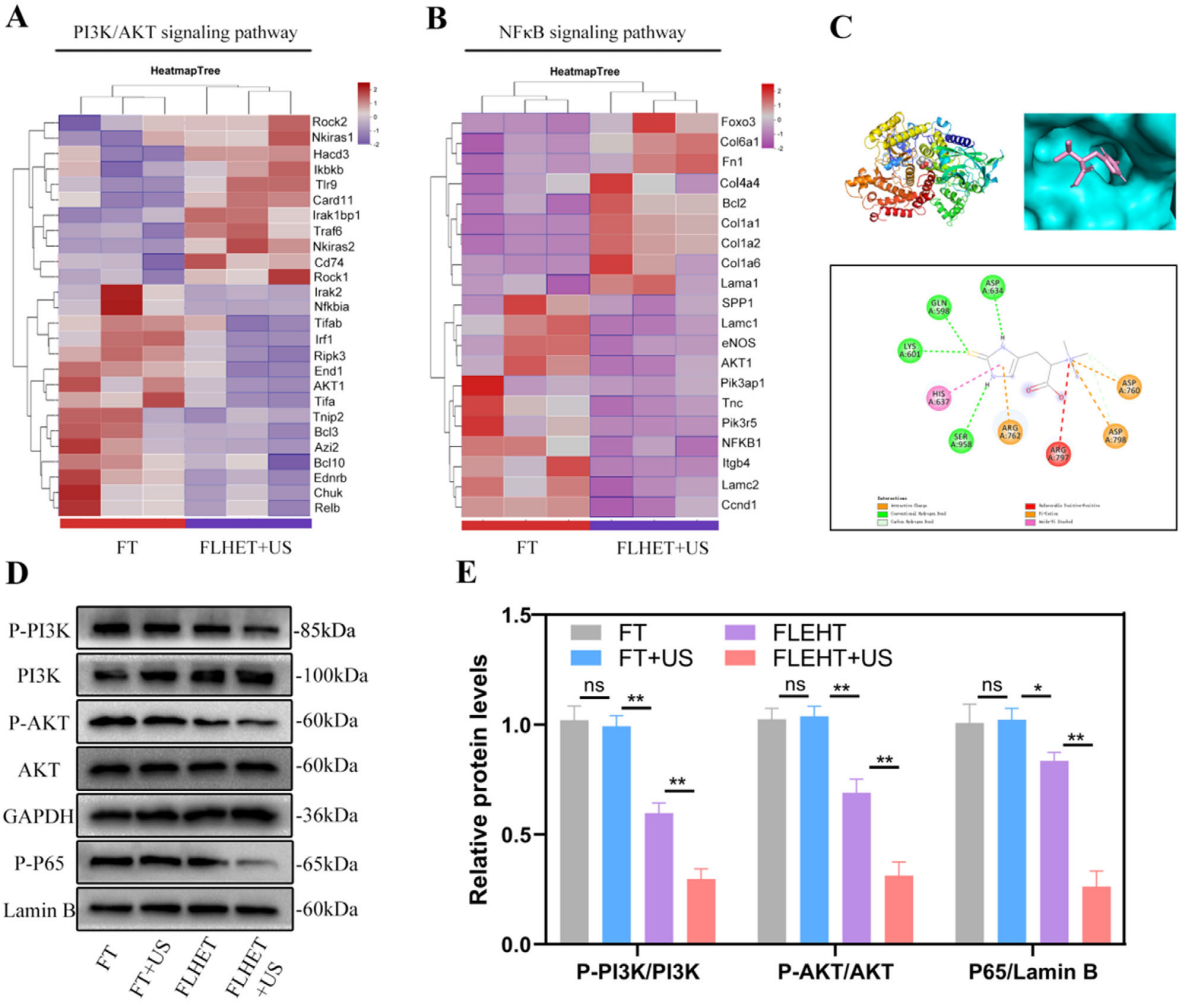
Effects of the “lever” hydrogels on the PI3K/AKT/NF‐κB signaling pathway. A,B) Heatmap of representative genes related to the PI3K/AKT and NF‐κB pathways. C) Molecular docking study of ET binding to the inhibitory pocket of PI3K. D) The protein expression of P‐PI3K, PI3K, P‐Akt, Akt, and intranuclear P‐P65 was detected by western blot in macrophages treated as described above. E) The ratios of P‐PI3K/PI3K P‐Akt/Akt, and P65/Lamin B were determined via ImageJ software. Values were indicated as mean±SD (*n* = 4). Significance between the two groups was calculated using one‐way ANOVA and the Tukey–Kramer multiple comparisons test. ns = no significance, ^*^ *p* < 0.05, ^**^ *p* < 0.01.

The PI3K/Akt pathway is activated during the inflammatory response and regulates the immune response.^[^ ^24^ ^]^ This pathway is an important upstream factor of NF‐κB and directly regulates the activity of NF‐κB, which is involved in inflammation in DFUs.^[^ ^25^ ^]^ According to the transcriptomic analysis of DFU tissues, FLHET hydrogels significantly activated genes involved in the “PI3K/AKT pathway” (GO: 0043491) and markedly decreased “I‐κB kinase/NF‐κB signaling” (GO: 0007249) on POD 7 (**Figure** **8**A,B). In addition, computerized molecular docking suggested a high affinity between ET and PI3K, which results in the formation of a stable complex. This complex interacts with the GLN598, LYS601, ASP634, SER958, ASP760, ASP798, HIS637, and ASP638 residues on PI3K, and the binding energy is −4.8 kcal mol^−1^, which is considerably less than the value of −4.25 kcal mol^−1^, suggesting that PI3K is the main target of ET (Figure 8C).

Based on the docking prediction, we subsequently performed a western blotting assay to confirm whether the FLHET hydrogels activate the PI3K/AKT signaling pathway. To mimic in vivo macrophage activation in chronic DFU wounds, we exposed RAW 264.7 cells to 12 h of LPS treatment prior to coincubation with US‐treated FLHET hydrogels. Compared with those in the control group, the P‐PI3K and P‐AKT levels in the FLHET+US hydrogel group were significantly increased, whereas nuclear p65 expression was significantly decreased (Figure 8D,E). In conclusion, US‐treated FLHET hydrogels can mitigate inflammation in chronic DFUs by modulating the PI3K/AKT/NF‐κB pathway to downregulate inflammatory cytokines.

### 2.9 Nanocomposite “Lever” Hydrogels Accelerated the Polarization of M2 Macrophages During Chronic DFUs

**Figure 9:**
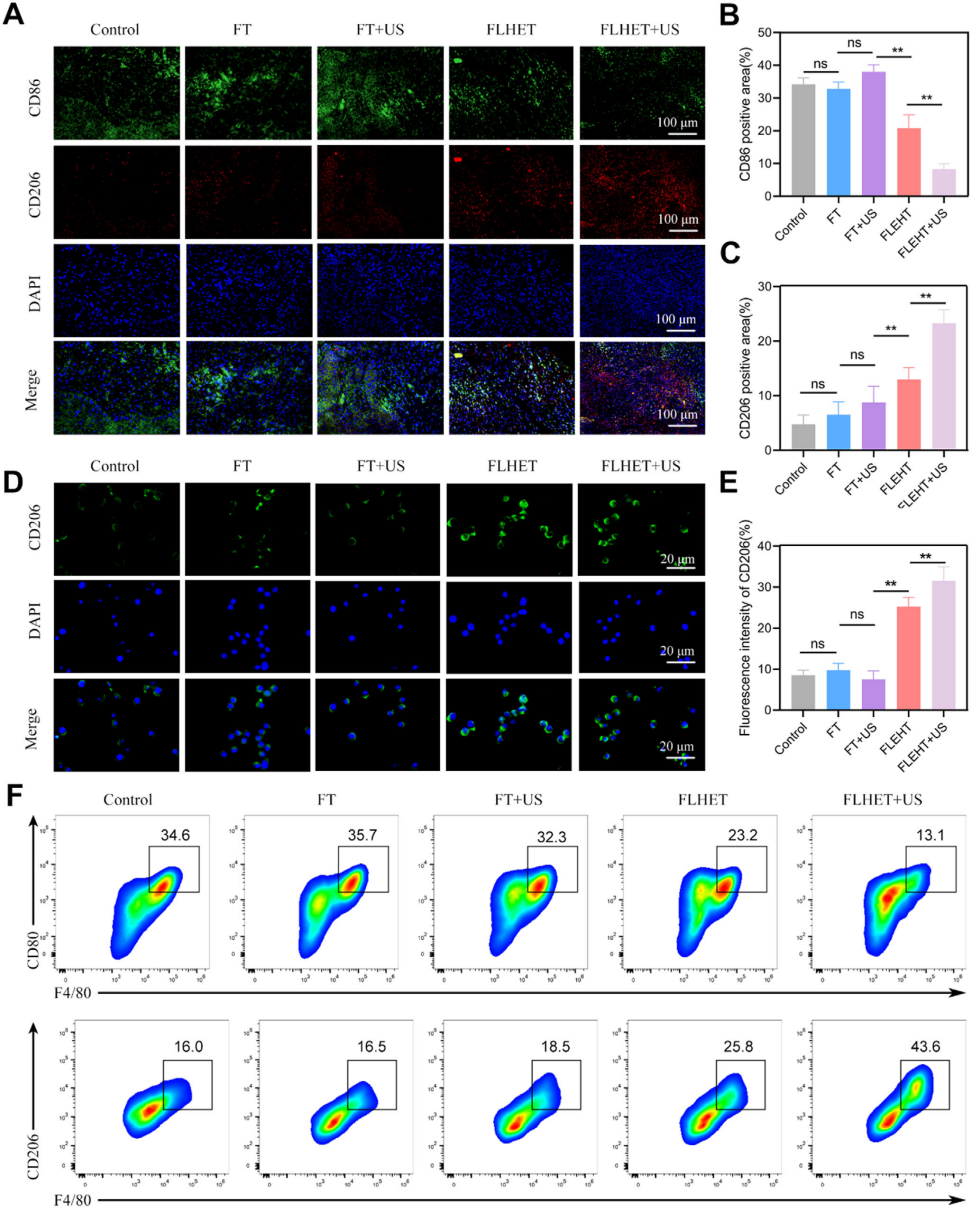
Effects of the “lever” hydrogels on M2 macrophage polarisation in vitro and in vivo. A) Dual immunofluorescence staining for CD86 (green) and CD206 (red) in wound tissue at POD 7. B,C) Quantification of the CD206‐ and CD86‐positive areas. The values are presented as the means±SDs (*n* = 4). D) Confocal images of immunofluorescence staining of RAW 264.7 cells for CD206 after 24 h of treatment in different groups. E) Quantitative analysis of the mean fluorescence intensity of CD206. F) Representative flow cytometry plots showing the presence of M1 (F4/80^+^, CD80^+^) and M2 (F4/80^+^, CD206^+^) macrophages after 24 h of treatment with different groups. Values were indicated as mean±SD (*n* = 4). Significance between the two groups was calculated using one‐way ANOVA and the Tukey–Kramer multiple comparisons test. ns = no significance, ^*^ *p* < 0.05, ^**^ *p* < 0.01.

Macrophages are the main immune cells involved in wound tissues and skin damage, and the transition of the proinflammatory M1 phenotype to the anti‐inflammatory M2 phenotype plays an important role in resolving inflammation and repairing wounds.^[^ ^26^ ^]^ To confirm whether macrophage transformation affects inflammation resolution following treatment with “lever” hydrogels, CD86 was used to stain M1 macrophages, whereas CD206 was used to label M2 macrophages. As revealed by an in situ immunofluorescence assay, FLHET+US hydrogels reduced the number of M1 (CD86) macrophages in DFU tissues but increased the number of M2 (CD206) macrophages (**Figure** **9**A–C). Moreover, in vitro immunofluorescence staining of RAW 264.7 cells was performed. The FLHET+US hydrogel group presented a pronounced transition of M1‐to‐M2 macrophages (Figure 9D,E). These fluorescence trends were further confirmed by flow cytometry data. Compared to the other groups, the FLHET+US hydrogel‐treated group had the lowest percentage of M1‐like macrophages expressed as F4/80^+^ and CD80^+^, and conversely, the highest percentage of M2‐like macrophages expressed as F4/80^+^ and CD206^+^ (Figure 9F). Collectively, these findings indicate that the nanocomposite “lever” hydrogels can modulate and promote M1‐to‐M2 macrophage polarization.

## 3 Conclusion

In summary, this study developed a novel in situ nanocomposite “lever” hydrogel to promote the healing of chronic refractory DFUs wounds. In the early infection stage, FLHET hydrogels produced a large amount of ROS under US treatment and exhibited excellent acoustic dynamic antimicrobial properties. Upon cessation of US, thrombin and fibrinogen are gelled in situ to fill the irregular wound. ET in the gel continuously scavenges excess ROS during the proliferative phase of the wound and induces macrophage polarization toward the M2 phenotype. Further mechanism exploration revealed that US‐treated FLHET hydrogels effectively reduced inflammation in DFUs wounds by activating the PI3K/AKT/NF‐κB signaling pathway. These results demonstrated that the novel nanocomposite “lever” hydrogels could solve the limitation that ordinary dressings could not achieve antibacterial and anti‐inflammatory simultaneously by reasonably regulating the ROS level based on the different demands for ROS at different stages of wound repair, and ultimately promote the comprehensive repair of wound tissues, which provides a new therapeutic strategy for the treatment of refractory DFUs‐infected wounds.

## 4 Experimental Section

### Ethical Approval

All animal experiments were carried out in accordance with the animal experimentation policy stipulated by the National Ministry of Health. All in vivo experiments were conducted after approval by the Animal Ethics Committee of the Shanghai Tenth People's Hospital. (SHDSYY‐2023‐1300061).

### Statistical Analysis

All experiments were performed as biological replicates at least three times. All data were expressed as mean values±standard deviation (SD). All the quantitative data in each experiment were evaluated and analyzed using one‐way ANOVA and the Tukey–Kramer multiple comparisons test for multiple‐group analysis in GraphPad Prism 9.0 software to evaluate the statistical significance of the variance. The “ns” presents no significance and a p‐value was used as a statistical significance threshold.

## Conflict of Interest

The authors declare no conflict of interest.

## Author Contributions

Y.S., S.L., and X.H. contributed equally to this work. H.X., H.Y., and B.H., designed and supervised the project. Y.S., L.S., X.H., J.Y., Y.Z., C.Z., Z.N., X.G., B.X., S.W., Y.Y., X.L., and L.S. performed the experiments, and collected and recorded data. All authors analyzed and interpreted the data. Y.S. wrote the manuscript. All the authors contributed to the discussion of the results and implications and edited the manuscript at all stages.

## Supporting information



Supporting Information

## Data Availability

The data that support the findings of this study are available from the corresponding author upon reasonable request.
