# Supplementary material for: Ultrasound‐Triggered Nanocomposite “Lever” Hydrogels with a Full Repair System Accelerates Diabetic Foot Ulcer Repair
Source: Adv Sci (Weinh). 2025 May 8;12(23):2500720. doi: 10.1002/advs.202500720 (PMC12199430; doi:10.1002/advs.202500720)
Supplement: Supplementary file 1 — Supporting Information [file ADVS-12-2500720-s001.docx]

Supporting Information

Ultrasound-triggered nanocomposite ‘lever’ hydrogels with a full repair system accelerates diabetic foot ulcer repair

Yuting Shen^1#^, Shaoyue Li^2#^, Xiaodong Hou^2#^, Jifeng Yu^1^, Yuli Zhu^1^, Chongke Zhao^1^, Zhiyuan Niu^3^, Xin Guan^1^, Bing Xiong^1^, Sirui Wang^1^, Yifei Yang^4^, Xiao Li^1^, Liping Sun^2^, Shengbo Wu^5^, Bin Huang^5^*, Haohao Yin^1^*, Huixiong Xu^1^*

**1. Materials and methods**

**1.1 Materials**

All chemicals were provided by Sigma-Aldrich, except where otherwise stated, and were used without further purification. HUVECs, RSC96, Raw264.7, Hacat and RS1 cells were obtained from Shanghai Zhong Qiao Xin Zhou Biotechnology Co., Ltd. Anti-CD86, anti-CD31, anti-α-SMA, anti-PGP9.5, anti-TNF-α, and anti-CD206 antibodies were purchased from Abcam (Cambridge, MA, USA). Antibodies against PI3K, P-PI3K, P-AKT, AKT, GAPDH, P-P65, IL-6, IL-1β, and Lamin B were obtained from Abcam (Cambridge, MA, USA). Antibodies against Collagen I (Col-1), were procured from Cell Signaling Technology (Beverly, MA, USA). Thrombin and fibrin were supplied by Beijing Solarbio Science & Technology Co., Ltd. Soya lecithin, cholesterol, and DSPE-Se-Se-PEG-NH2 were obtained from Shanghai Ponsure Biological Technology Co., Ltd. Ultrasonic apparatus (Chattanooga 2776) were procured from Tejio Medical Equipment Trading Co., Ltd. (Shanghai, China). All other reagents were bought from domestic providers and utilized as received.

**1.2 Synthesis and characterization**

1.2.1. Preparation of LHET by the thin-film dispersion-sonication method

A mixed lipid mixture was obtained by dissolving 8.5 mg of soy lecithin, 1 mg of cholesterol, and 2.5 mg of DSPE-Se-Se-PEG-NH2 in 6 mL of chloroform. HMME (5 mg/mL) was sufficiently blended with DMF. Subsequently, 60 µL of HMME was added to the abovementioned lipid mixture. Phospholipid films were obtained by evaporation with a rotary evaporator at 50 °C for 30 min. Next, 5.38 mL of deionized water, 20 µL of ET (5 mg/mL), and 600 µL of thrombin (1000 U/mL) were added to this flask for film dissolution. Nanocomposites (liposome@HMME-ET-Thrombin, LHET) were obtained by sonication using an ultrasonic cleaner for 5 min; the solution was hydrated at 37 °C for 1 h, cooled to 25 °C, and then subjected to probe sonication for 2 min (600 W, working 3 s/interval 1s) under an ice bath. Additionally, liposome@HMME-Thrombin (LHT) with no ET, liposome@HMME-ET (LHE), liposome@HMME (LH), and pure liposome (Lip) with no thrombin were prepared via the same synthesis steps for further analysis.

1.2.2. TEM and DLS analyses

To prepare samples for topographic analysis, we added these nanocomposite solutions to a copper grid, followed by drying with a light heat lamp. A JEMe1400 (HITACHI, Japan, 120 kV) transmission electron microscope was then used to obtain images. Z-potential was assessed with a Zetasizer Nanoseries (Nano ZS90, Malvern). The hydrodynamic diameter of the nanocomposite was determined via DLS. DLS was conducted to test nanocomposite stability at 37 °C over a 7-day period. To confirm the ROS-induced rupture of LHET NPs following US treatment, TEM was conducted to observe LHET and Lip NPs (with no HMME sensitizer) following 3 min of US exposure.

1.2.3. UV spectroscopy

To qualitatively analyse the HMME loading of the NPs, we obtained typical absorption peaks for the liposomes, LH, LHE, LHT, and LHET at 300–500 nm with a UV-vis spectrophotometer (Thermo Scientific, USA).

1.2.4. Raman spectroscopy

To conduct a qualitative analysis of the ET loading of the NPs, typical absorption peaks for LHET were detected on the basis of the characteristic Raman scattering spectrum at 484 nm. In addition, for the relative quantitative analysis of ET content in each organ and skin tissue sample, the typical absorption peaks of ET in each sample were detected according to the characteristic spectrum of Raman scattering spectrum at 484 nm and the intensity was recorded.

1.2.5 *In situ* nanocomposite hydrogel synthesis

Fibrinogen (100 mg) was blended with saline (1 mL) in a 37 °C shaker (Bluepard, Shanghai) for a 1 h period until complete dissolution. BCA was then conducted to quantify 100 UI/mL thrombin in LHET NPs. Next, the fibrin hydrogel precursors of fibrinogen were completely mixed with LHET NPs (primarily thrombin, FLHET) at a 1:1 volumetric ratio. The resulting solution was then subjected to US treatment to induce gelation with a US device (Chattanooga Group, USA) at 1 W cm^–2^ power, 1 MHz frequency, and 50% duty cycle for 3 min.

1.2.6. Drug release profile

To evaluate the ET release profile in fibrin-based *in situ* hydrogels, we ultrasonicated samples at 0, 4, 8, 12, and 24 days and then placed these hydrogels in a dialysis bag (MWCO = 7000 Da). After mixing in 50 mL centrifuge tubes containing 10 mL of DPBS on a shaking bed (37 °C) at 120 rpm, aliquots of the medium (200 mL) were obtained from the centrifuge tubes at specific time points. Next, an identical amount of PBS was added to the medium. Raman spectroscopy was used to quantify the ET concentrations at diverse time points, as described previously.

1.2.7. SEM analysis

Hydrogel samples (FT+US, FLHT+US, and FLHET+US hydrogels) were immersed in phosphate buffer solution (PBS, pH 7.4) and placed in a thermostatic shaker (25 °C) with continuous shaking at 100 rpm for 24 h to reach swelling equilibrium. The swelling end point was determined by mass method (error of swelling ratio <3% for three consecutive measurements). The equilibrated samples were transferred to an ultra-low temperature refrigerator at -80°C for pre-freezing for 24 hours to ensure complete solidification. The pre-frozen samples were placed in a freeze dryer (xx model) and lyophilised at -60°C cold trap temperature and 0.1 mBar vacuum for 48 hours to completely remove water. After the freeze-drying was completed, a 5 nm thickness Au-Pd alloy layer was uniformly coated on the sample surface using an ion sputtering apparatus (sputtering current 20 mA for 120 s) to eliminate the charge accumulation effect. Subsequently, morphological characterisation was carried out using a field emission scanning electron microscope (SEM, JMF-7500F, Japan) at an accelerating voltage of 5 kV.

1.2.8 ROS measurement

ROS produced through the nanocomposite hydrogels were analyzed with a DPBF probe, with pure fibrin hydrogels used as a control. Briefly, fibrinogen (100 mg) was sufficiently blended into 1 mL of PBS containing 10 mM DPBF. Later, fibrinogen was added to LHET (100 UI/mL thrombin) at a ratio of 1:1 (v:v), followed by ultrasonic treatment to induce gelation. Following the immersion of the hydrogels in 2 mL of PBS, the DPBF absorbance values were detected with a multifunctional microplate reader at 0, 2, 4, 6, 8, 10, and 20 min and at 410 nm.

**1.3 *In vitro* cell analyses**

1.3.1. CCK-8 assay

To assess the activity of US alone on cells critical for wound healing, HUVECs, RSC96, fibroblasts cells (RS1), Raw264.7, and keratinocyte migrating cells (Hacat) were first treated with US for 1-5 min each, and then inoculated in 96-well plates at 5× 10^3^ cells per well. US exposure was then conducted at 1 W cm^-2^ power with 50% duty cycle and 1 MHz frequency. After 24 h of incubation in complete medium (100 µL) in 96-well plates, freshly prepared medium containing 10% CCK-8 solution was added to replace the medium. Following 2 h of coincubation in the cell culture incubator, the absorbance values of the supernatants were detected with a multifunctional microplate reader at 450 nm. In addition, to evaluate the optimal biosafety concentrations of FT and FLHET, HUVECs, RSC96, RS1, Raw264.7, and Hacat cells were co-cultured with different concentrations of FT and FLHET without US, respectively, and the viability of the wound healing-critical cells was measured after 24 h of co-incubation in the cell culture incubator, and by CCK8 as described previously.

1.3.2. *In vitro* biocompatibility of nanocomposite hydrogels

For the CCK-8 assay, HUVECs, RSC96, and RS1 (1 × 10^5^/hydrogel scaffold) were inoculated into the hydrogels. Briefly, the cells were resuspended in 100 mg mL^–1^ fibrinogen solution at a cell density of 2 × 10^7^ cells/mL. Fibrinogen and different NPs (100 UI/mL, thrombin) were introduced into cylindrical moulds (4 mm × 2 mm) at a ratio of 1:1 (20 mL). The precursor solution was then subjected to 3 min of ultrasonic treatment at 1 W cm^–2^ to form the nanocomposite hydrogels. We classified these *in situ* nanocomposite hydrogels into the following 4 groups: (1) FT, (2) FT+US, (3) FLHET, and (4) FLHET+US. Following gelation, we cultivated the cell-scaffold constructs in DMEM for subsequent analysis. Next, a 1 h UV light treatment was conducted to sterilize the fibrinogen precursor.

1.3.3. AM/PI staining

Following US exposure, the nanocomposite hydrogels were soaked in complete medium supplemented with high-glucose DMEM containing 10% foetal bovine serum for various durations (0, 24, 48, and 72 h). The biocompatibility of the obtained extract mixture was subsequently analyzed. Cell viability was tested via the LIVE/DEAD assay (Beyotime, Shanghai, China).

1.3.4. Antibacterial performance

In order to evaluate the antimicrobial properties of the hydrogels, FLHET+US hydrogels were evaluated using Escherichia coli and Staphylococcus aureus as bacterial models. Briefly, Staphylococcus aureus and E. coli were incubated overnight in Luria-Bertani (LB) broth at a shaker temperature of 37 °C. Then FT, FT+US, FLHET+US, and FLHET+US hydrogels were added to the petri dishes. 200 pL of bacterial suspension (106 CFU/mL) was added to the surface of the hydrogels of different compositions. Then the antimicrobial properties of the hydrogels were observed after the above hydrogels corresponded to sonication or no sonication.

1.3.6. Scratch assay

A scratch assay was conducted in a 12-well plate. After 95% cell density/well was reached by using the extract mixture (48 h), a 200 µL pipette tip was used to make a scratch. Images were visualized with a phase contrast microscope and analyzed with ImageJ software version 5.0.

1.3.6. Tube formation assay

HUVECs (2.5 × 10^4^/well) were inoculated into the extract mixture (48 h) in 96-well plates previously coated with Matrigel Basement Membrane Matrix (70 µL, BD Biosciences, CA, USA). A microscope was used to detect tube formation after 6 h of incubation. The data are expressed as the means ± SEMs from 3 separate assays.

1.3.7. ELISA of inflammatory cytokines

The conditioned cell culture medium was obtained after incubation and subjected to centrifugation for 20 min at 14,000 rpm. The levels of inflammatory factors, including IL-1β, IL-6, and TNF-α, were detected by corresponding ELISA kits in accordance with specific instructions.

1.3.8. Immunofluorescence

The cells were inoculated into 6-well plates with coverslips and coincubated with US-based hydrogels for 12 h prior to 24 h of stimulation with LPS. After 20 min of fixation with 4% paraformaldehyde, the cells were subjected to 10 min of permeabilization with 0.5% Triton X-100 and blocked for 1 h with 10% goat serum at room temperature. Following overnight incubation at 4 °C with primary antibodies (anti-CD206), the samples were rinsed and further probed with Alexa Fluor®488-labeled secondary antibodies for 1 h at room temperature. After three washes, the slides were counterstained with DAPI for 1 min. The slides containing fluorescent mounting medium were covered with coverslips. A fluorescence confocal microscope (FV3000, Olympus, Japan) was used to acquire images.

**1.4. *In vivo* analysis**

1.4.1. Diabetic foot ulcer rat model

Male adult Wistar rats aged 12–16 weeks and weighing 180–220 g were used to establish type I diabetic model rats^[1]^. Briefly, each normal Wistar rat was intraperitoneally administered STZ (1%; dose: 80 mg/kg) following 12 h of fasting. An autoanalyzer (AccuCheck Active, Roche Diagnostics) was used to determine the fasting blood glucose levels of the rats on days 3, 5, and 7. The successful generation of a type I diabetic rat model was based on the following criterion: blood glucose > 16.7 mM for 7 consecutive days. After the diabetic rat model was established, we created full-thickness diabetic wounds on the left side of each rat via a punch biopsy device (diameter, 10 mm). The animals were then randomized into 4 experimental groups (n = 10) for FT, FT+US, FLHET, and FLHET+US hydrogel treatments (100 µL, containing an ET dose of 15 mg/kg and an HMME dose of 8 mg/kg). The wounds were subsequently covered with gauze to prevent foot licking by the rats. US exposure/unexposure was then conducted at 1 W cm^-2^ power with 50% duty cycle and 1 MHz frequency for 3 min on days 0, 3, 7, 14, and 21, respectively. Foot ulcer wound photos were obtained at specific time points to observe wound healing. All animal experiments were conducted in accordance with the protocols stipulated by the policy of the National Ministry of Health and approved by the Animal Ethics Committee of the Shanghai Tenth People's Hospital (SHDSYY-2023-1300061).

1.4.2. Biodegradation evaluation

Thirty-two SD rats were randomly divided into four groups. Following general anaesthesia, we created two 2 cm skin incisions on the back, followed by blunt separation of the subcutaneous tissue. We then implanted 200 µL of FT, FT+US, FLHET, or FLHET+US hydrogels in the subcutaneous pocket, with the nontreatment group used as the control. At 1, 3, and 6 weeks postoperatively, digital photos showing the residual hydrogels were obtained (denoted by a ruler in the field of view).

1.4.3. Dihydroethidium (DHE) staining

The DHE probe was used to detect reactive oxygen species levels in tissues^[2]^. Firstly, the DFU wound tissue was dehydrated in a 30% sucrose solution, followed by placing the dehydrated tissue on filter paper to remove the water completely. Next, the tissue samples were covered with OCT embedding agent and subsequently cooled until the OCT was completely solidified. The solidified tissue samples were cut into 20 μm frozen sections. After thawing, the sections are treated with an autofluorescence quencher for 5-10 minutes. The DHE stock solution is diluted to the appropriate concentration. The solution is then quickly pipetted onto the tissue samples and incubated in the staining solution at 37°C for 30 minutes. Finally, the tissue slides are washed in PBS for 3-5 min at room temperature and imaged using a fluorescence microscope.

1.4.3. Histology, immunohistochemistry, and immunofluorescence assay

At 3, 7, 14, and 21 days post-treatment, we obtained wound samples for hematoxylin-eosin (HE) staining to evaluate wound inflammation and epidermal regeneration^[3]^. We also performed Masson staining to evaluate collagen deposition in the wound bed^[4]^. Anti-Col-I, anti-IL-1β, anti-IL-6, and anti-TNF-α antibodies were used for the immunohistochemistry. Immunofluorescence staining was performed with the following antibodies: anti-CD86, anti-CD206, anti-PGP9.5, anti-CD31, and anti-α-SMA.

1.4.4. Measurement of the mechanical sensitivity and thermal sensitivity.

Measurements were conducted at 1, 2, 4, and 6 weeks postoperatively, as described previously^[5]^. Briefly, to measure mechanical sensitivity, the rats were placed at the bottom of individual cages with wire mesh. The area close to the incision was vertically stimulated via Von Frey fibres. After slight bending of the fibres for 4–6 s or when the rats started to show behavioural reactions, the stimulation was terminated. The paw mechanical withdrawal threshold was measured twice in each rat at 15-s intervals. The rats were placed on a glass plate to measure thermal sensitivity. Following 30 min of environmental adaptation, irradiation light was applied to stimulate the incision on the hind paw of each rat. After the rat initiated paw withdrawal or after 20 s of irradiation, we terminated the irradiation and recorded this duration as the threshold of thermal pain. The thermal sensitivity value was averaged from three measurements in every rat every 5 min.

1.4.5. Western blotting assay

The skin was collected from the wound edges and lysed in RIPA buffer after whole skin samples were shredded with surgical scissors. A BCA protein assay kit was used to measure the protein content. Protein separation was conducted via 12% polyacrylamide gel electrophoresis, followed by transfer onto polyvinylidene difluoride membranes. Next, 5% defatted milk (5%, w/v) was applied to block the membranes for a 2-h period at 25 °C. The membranes were subsequently rinsed with TBST solution, followed by overnight incubation at 4 °C with primary antibodies, including anti-VEGF (1:1000), anti-Sirt1 (1:1000), anti-Nrf2 (1:1000), and anti-GAPDH (1:1000), followed by another 2-h incubation with secondary antibodies at ambient temperature. An ECL Plus reagent kit was used for band visualization. Finally, the blot signal intensities were analyzed with Image Lab 3.0 software (Bio-Rad, Hercules, CA, USA).

1.4.6. Molecular docking

ChemBioDraw was used to draw the molecular structure of ET, and ChemBio3D software was used to minimize the energy of ET. We acquired the P3K 3D crystal structure (code: 7Z75) from the Protein Data Bank (<https://www.rcsb.org/>). AutoDock Vina software was used to analyse protein-ligand docking by using default parameters. PyMOL (version 1.7.6) was used to visualize the minimal energy conformation for docking and 3D images. 2D images were created by Ligplot + software.

1.4.7. RNA-seq and mechanism validation

On postoperative day 7, tissue samples of DFU wounds were collected from three randomly selected modelled rats from the FT and FLHET+US groups. Total RNA was extracted from diabetic wound tissues with TRIzol reagent (Invitrogen, USA), based on the protocol by Zhou et al^[6]^. Residual DNA was eliminated through DNase I treatment. RNA purity was assessed by measuring the A260/A280 absorbance ratio with a Nanodrop™ One spectrophotometer (Thermo Fisher Scientific, USA). The integrity of RNA was verified via 1.5% agarose gel electrophoresis. Finally, RNA concentrations were determined using a Qubit 3.0 Fluorometer paired with the Qubit RNA Broad Range Assay kit (Life Technologies, Q10210, USA).

Two micrograms of RNA samples were used for stranded RNA sequencing library preparation using the KCTM Stranded mRNA Library Prep Kit for Illumina (Catalog No. DR08402, Wuhan SeqHealth Co., Ltd. China), according to the manufacturer's instructions. PCR products of 200–500 bp were enriched, quantified, and sequenced on a NovaSeq 6000 sequencer (Illumina) with the PE150 model. Raw reads were cleaned and aligned to the mRatBN7.2 genome using the TopHat2 software^[7]^. Uniquely mapped reads were used to calculate the read number and fragments per kilobase of transcript per million fragments mapped (FPKM) value for each gene. EdgeR software was used to measure the FPKM values and analyze the DEGs using RNA-seq data. The genes were analyzed and reported as fold change (fold change ≥2 or ≤ 0.5) of the control and a false discovery rate (FDR <0.05)^[8]^. Heatmaps were generated using the TBtools software (https://github.com/CJ-Chen/TBtools/releases). Gene Ontology (GO) and enriched KEGG pathway analyses were carried out using the KOBAS 2.0 server to predict the gene function and calculate the frequency distribution of functional categories^[9]^. The hypergeometric test and Benjamini-Hochberg FDR controlling procedure were used to define the enrichment of each pathway (corrected p-value <0.05).

1.4.8. Statistical analysis

All experiments were performed as biological replicates at least three times. All data were expressed as mean values±standard derivation (SD). All the quantitative data in each experiment were evaluated and analyzed using one-way ANOVA and Tukey-Kramer multiple comparisons test for multiple-group analysis in GraphPad Prism 9.0 software to evaluate the statistical significance of the variance. The “ns” presents no significance, and a p-value of was used as a statistical significance threshold.

**Supplementary Figures**


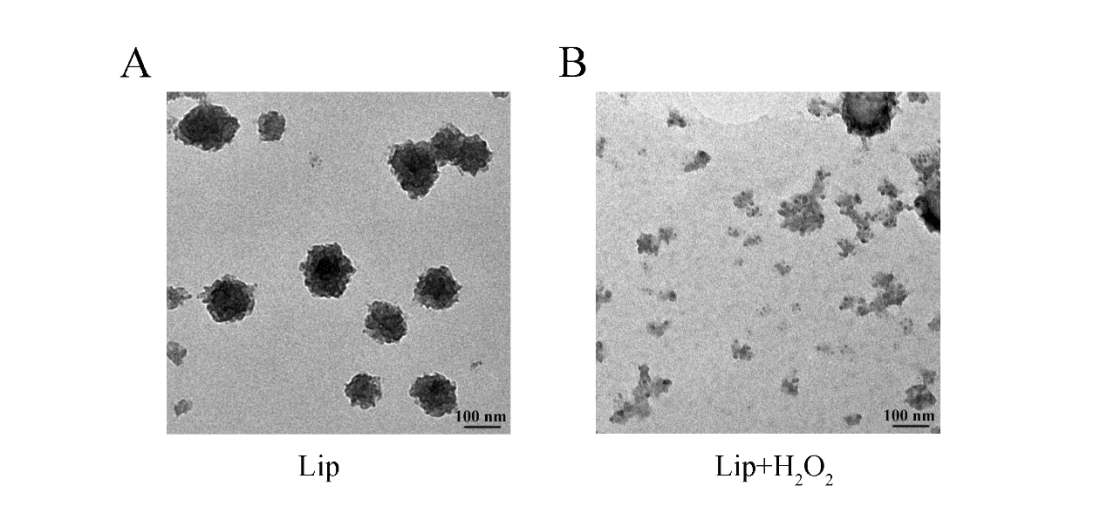


**Figure S1.** The rupture of Lip NPs triggered by ROS under H_2_O_2_ treatment. (A) TEM image of Lip NPs. (B) TEM image of Lip NPs with US treatment.


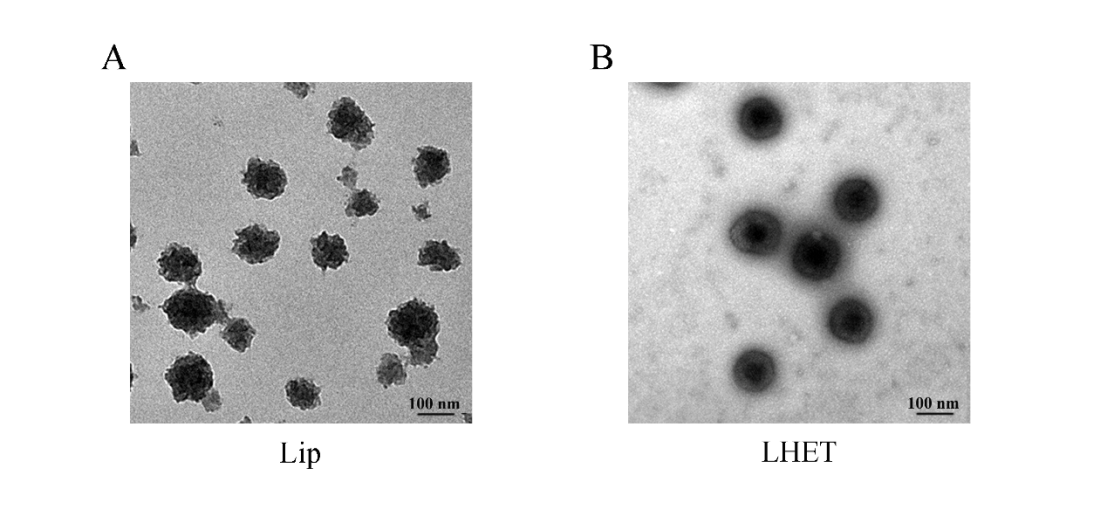


**Figure S2.** Representative TEM images of Lip and LHET. (A) TEM image of Lip NPs. (B) TEM image of LHET NPs.


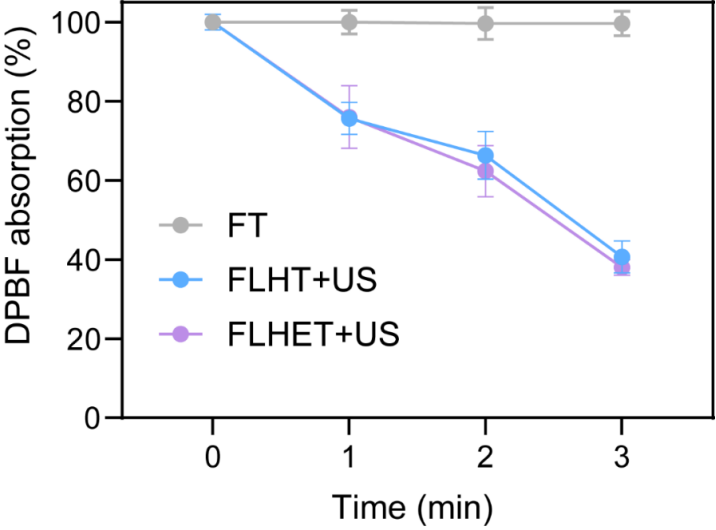


**Figure S3.** Absorption value of the DPBF probe after incubation with FT, FLHT+US and FLHET+US hydrogels under different US durations.


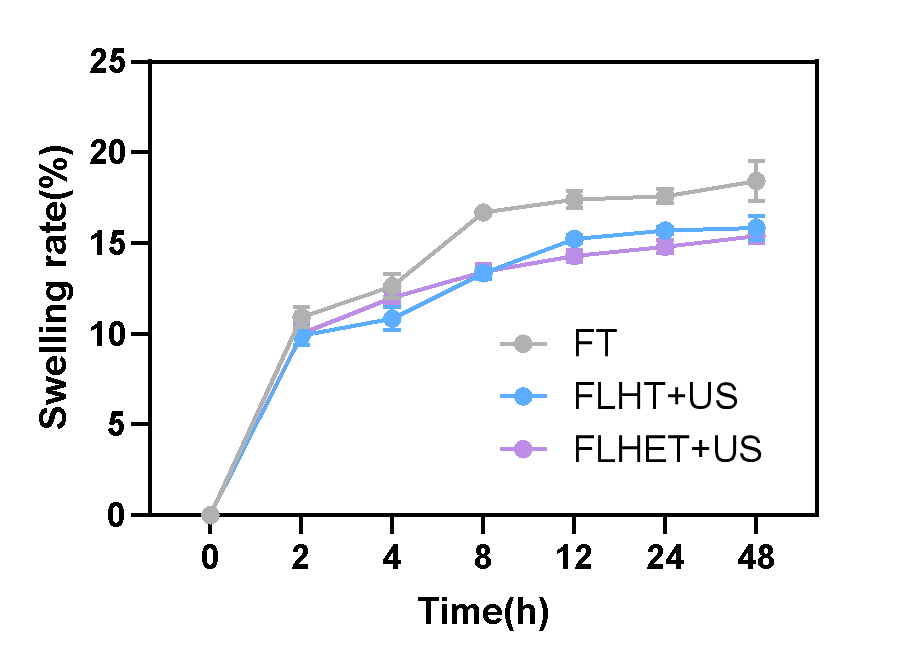


**Figure S4.** The average swelling rate of FT, FLHT+US and FLHET+US hydrogels.

**
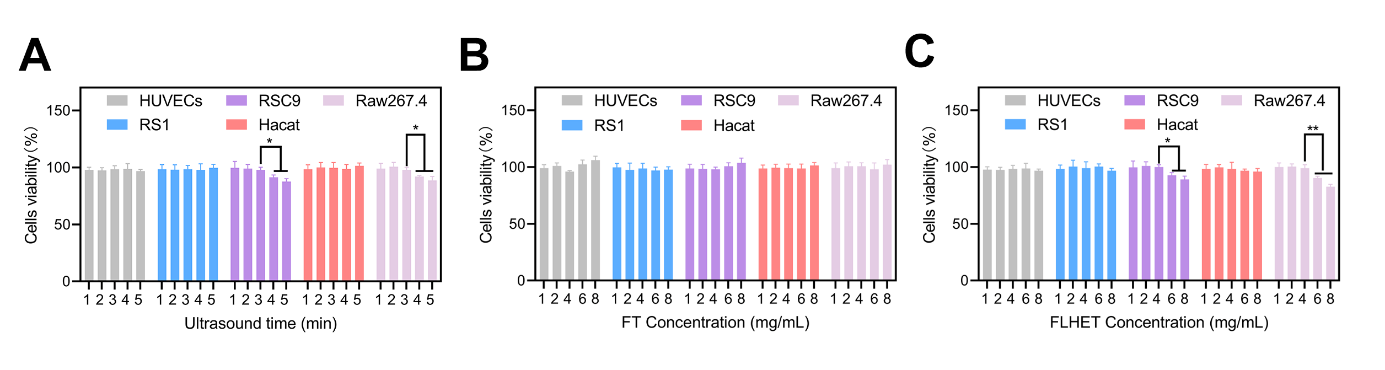
**

**Figure S5. The biological safety evaluation of the nanocomposite ‘lever’ hydrogels.** (A) Cell viability of HUVECs, RSC96, Raw264.7, RS1 and Hacat at different US (1 W cm^-2^) duration of treatment. (B) Cell viability of HUVECs, RSC96, Raw264.7, RS1 and Hacat after 24 h treatment with different concentrations of FT hydrogels. (C) Cell viability of HUVECs, RSC96, Raw264.7, RS1 and Hacat after 24 h treatment with different concentrations of FLHET. Values were indicated as mean ± SD (n=5). Significance between two groups was calculated using one-way ANOVA and Tukey-Kramer multiple comparisons test. ns=no significance, **P* < 0.05, ***P* < 0.01.


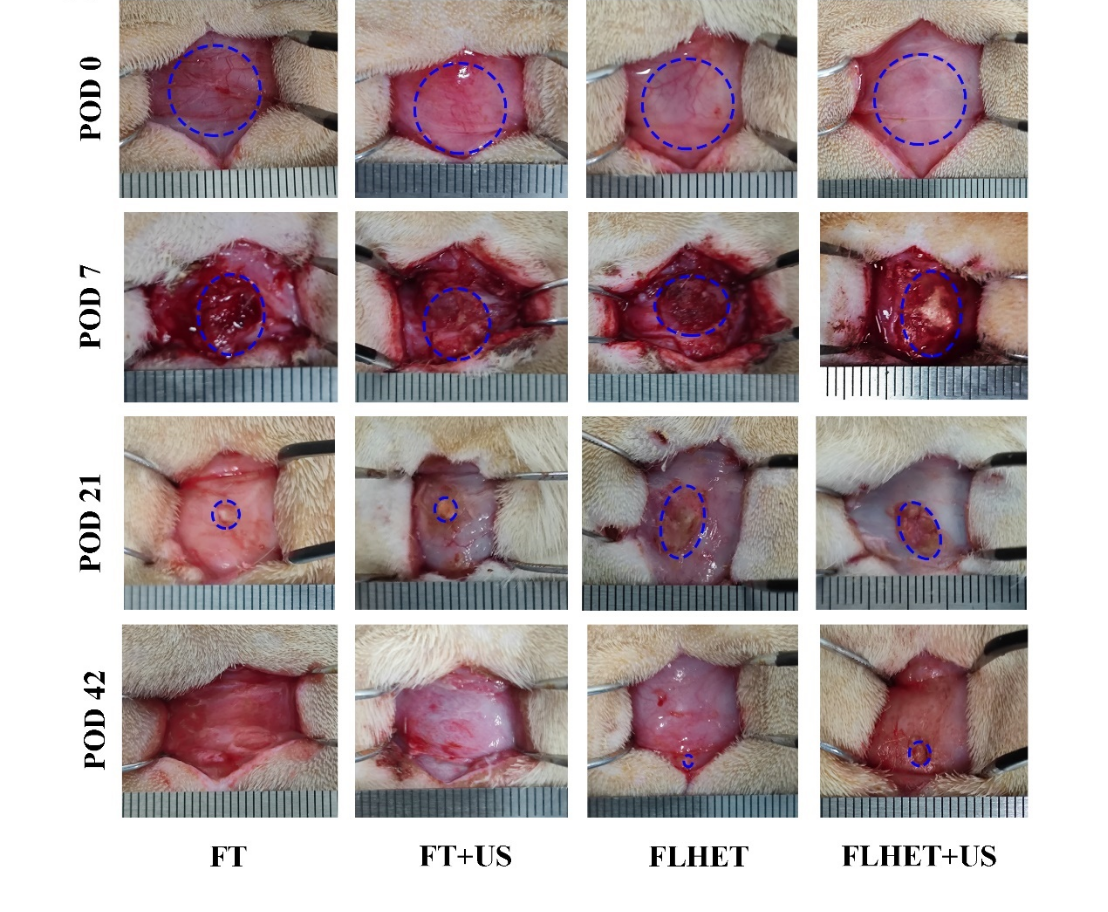


**Figure S6.** The *in situ* nanocomposite ‘lever’ hydrogels gradually degraded over time in the rat subcutaneous implant model (n=4).


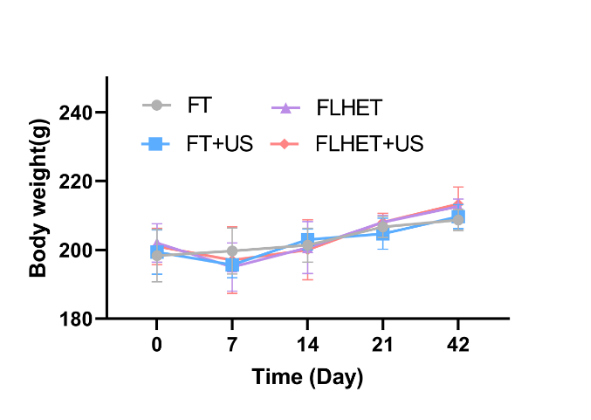


**Figure S7.** Quantitative analysis of body weight for each group on days 0, 7, 14, 21 and 42. The values are presented as the means±SDs (n = 4).


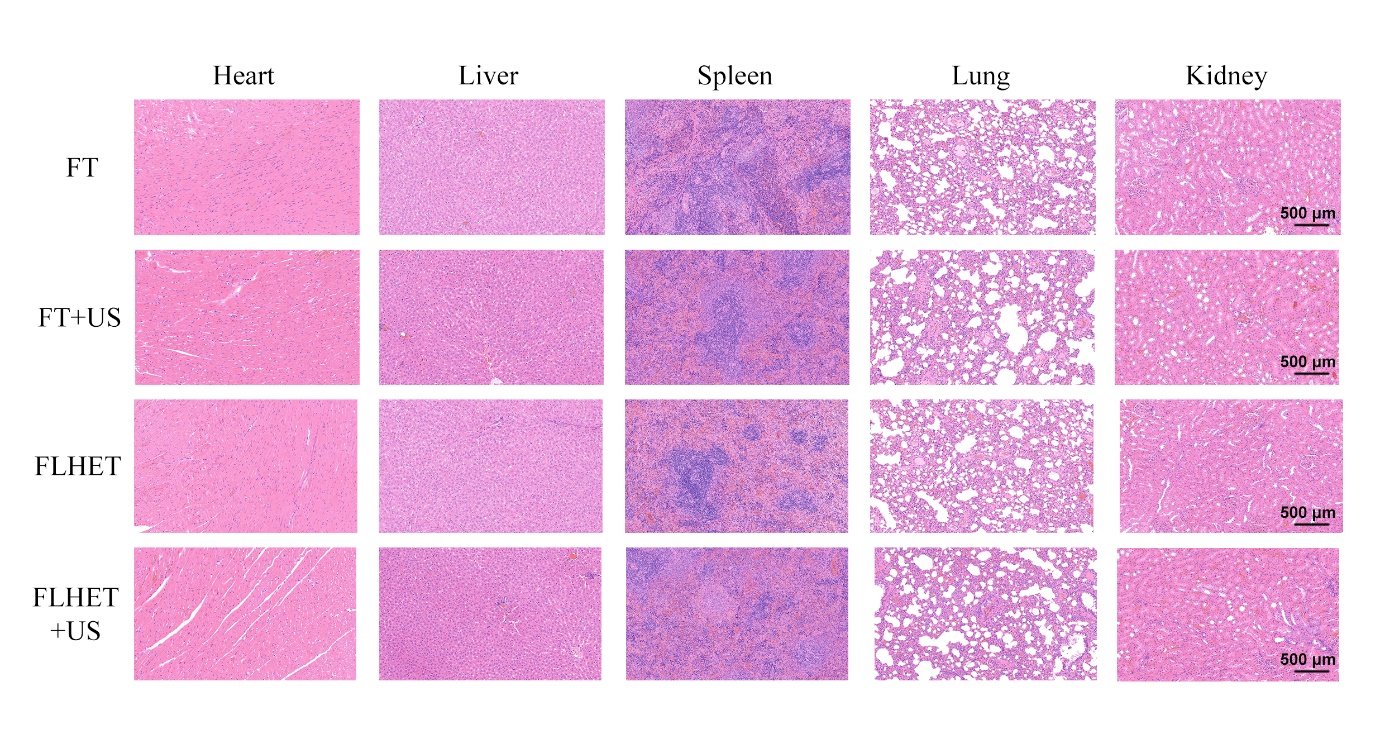


**Figure S8.** Major organs were collected for biosafety assessment after different treatments and for the formation of *in situ* nanocomposite hydrogels for 21 days (n=4). (Scale bar: 500 μm).


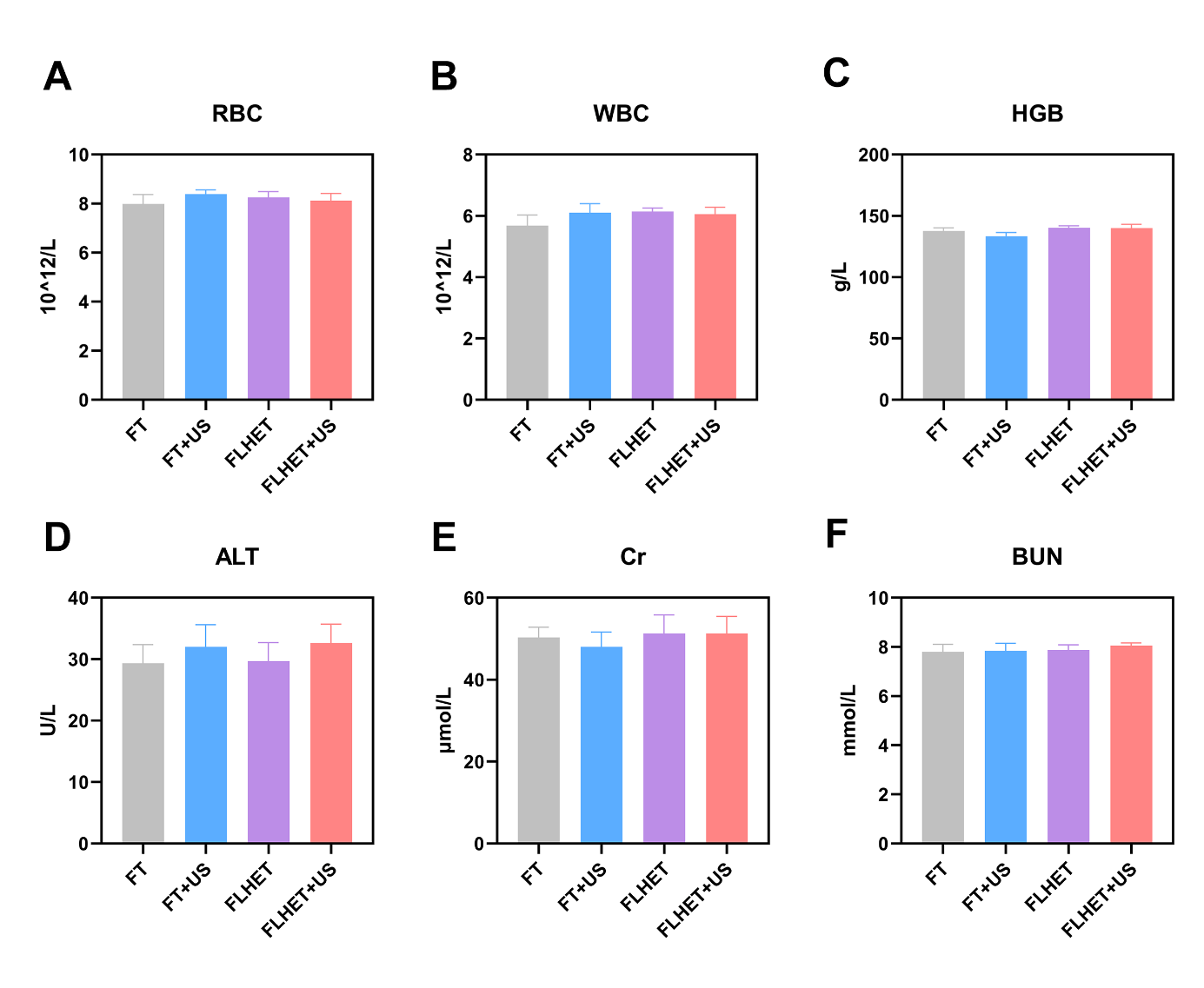


**Figure S9.** (A-C) After 21 days of treatment with different nanocomposite hydrogels, peripheral blood was collected and evaluated for routine blood tests (n=4). (D-E) After 21 days of treatment with different nanocomposite hydrogels, peripheral blood was collected and evaluated for liver and renal function (n=4).


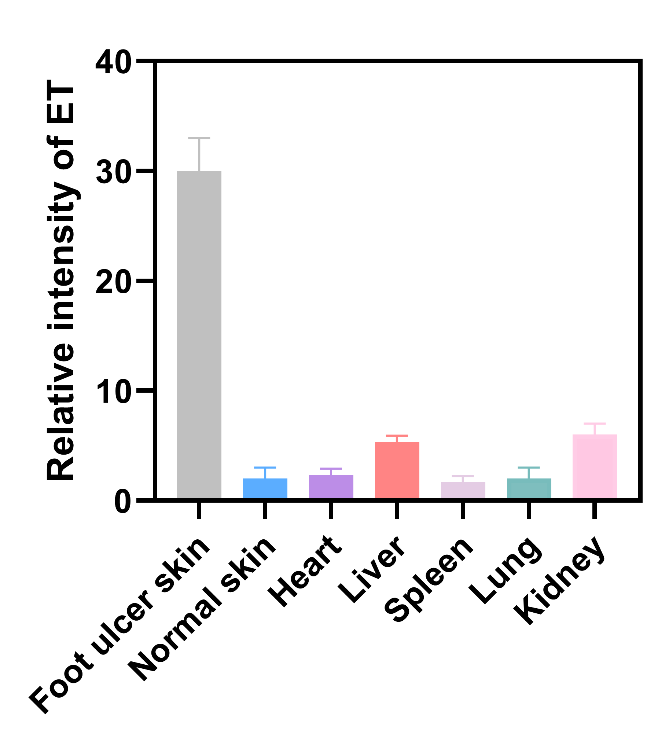


**Figure S10.** After 7 days of treatment with different nanocomposite hydrogels, samples of rat vital organs, DFU trauma skin, and normal skin tissue were collected and the relative amount of ET within each sample was measured by Raman spectroscopy (n=4).


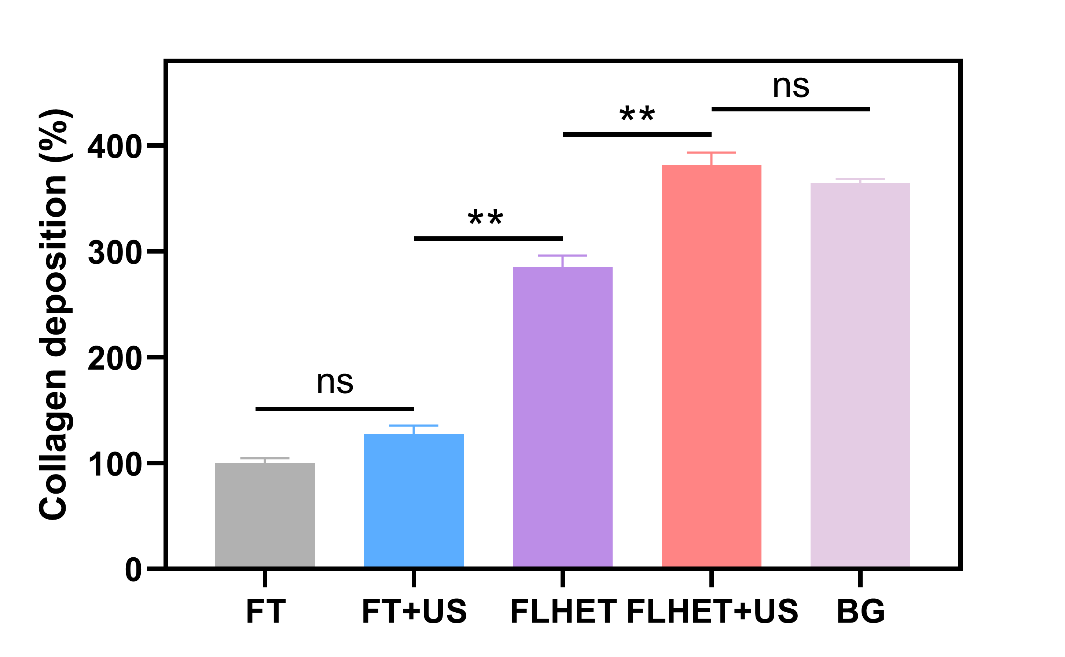


**Figure S11.** Quantitative statistics of collagen deposition rates after different treatments. Values were indicated as mean±SD (n=4). Significance between two groups was calculated using one-way ANOVA and Tukey-Kramer multiple comparisons test. ns=no significance. ***P* < 0.01.


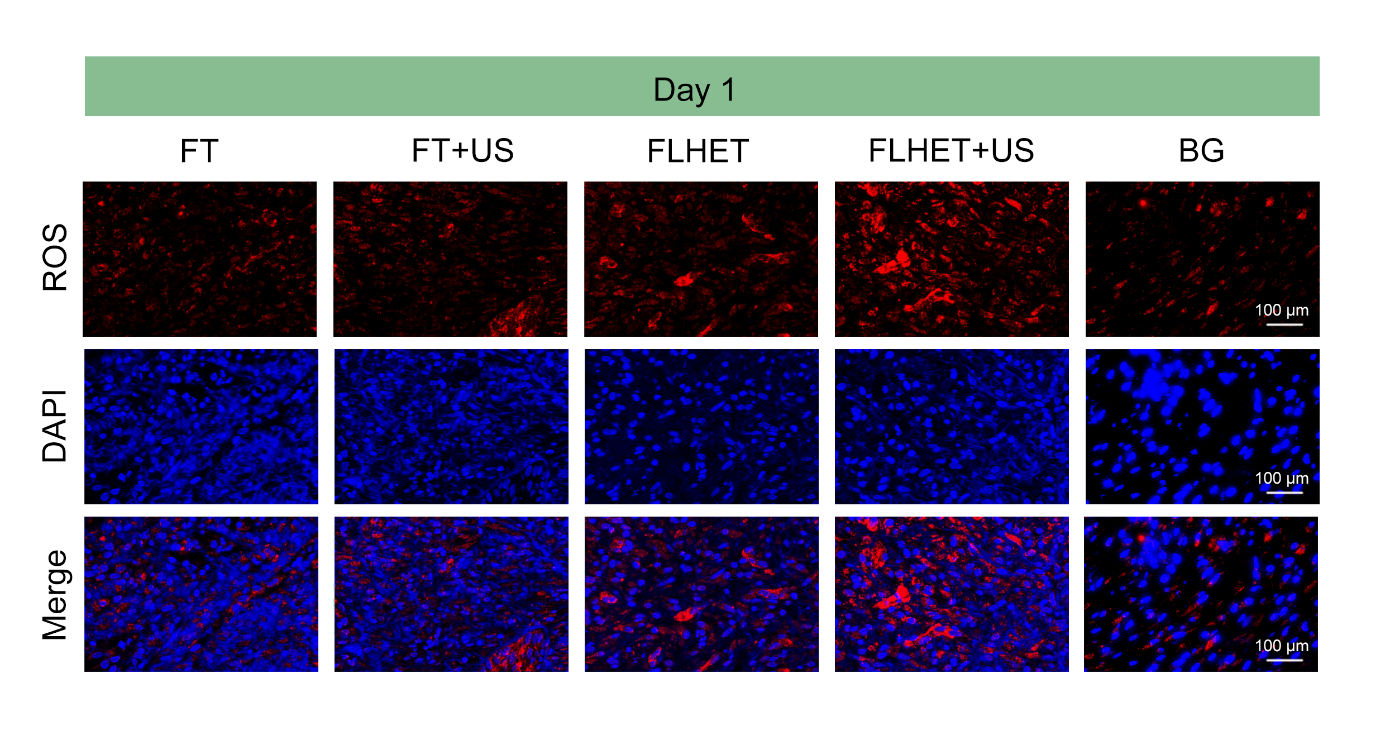


**Figure S12.** ROS levels in tissues of DFU wounds on day 1 after different hydrogels treatments (n=4).


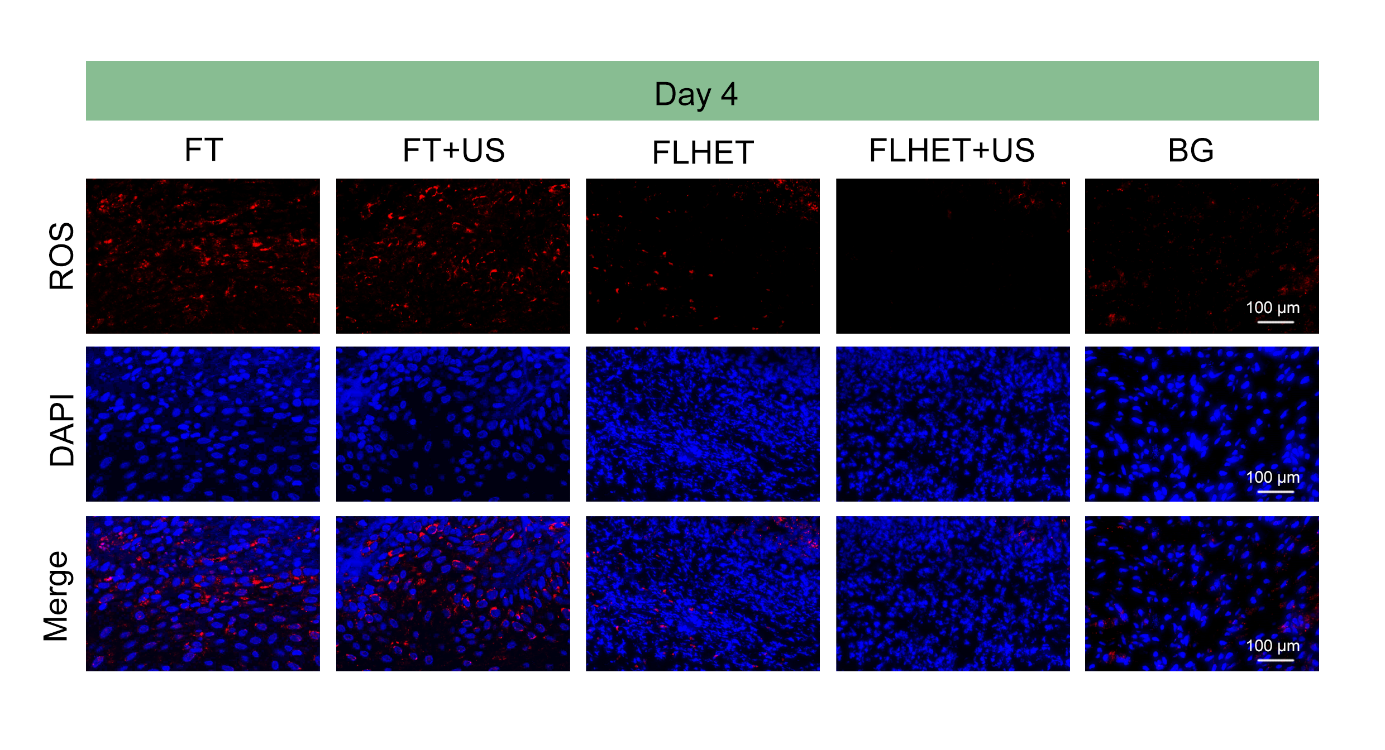


**Figure S13.** ROS levels in tissues of DFU wounds on day 4 after different hydrogels treatments (n=4).


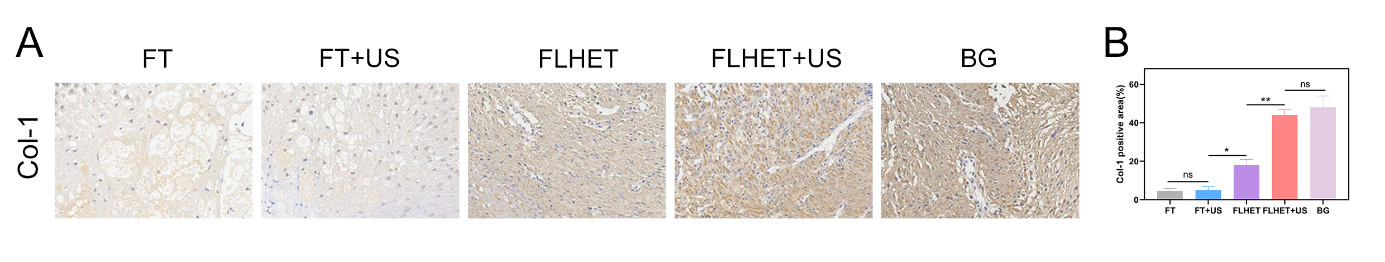


**Figure S14.** (A, B) Immunohistochemical images of Col-1 expression levels in DFU wound tissues on day 4 after different hydrogel treatments and their quantitative analysis. Values were indicated as mean±SD (n=4). Significance between two groups was calculated using one-way ANOVA and Tukey-Kramer multiple comparisons test. ns=no significance, **P* < 0.05, ***P* < 0.01.


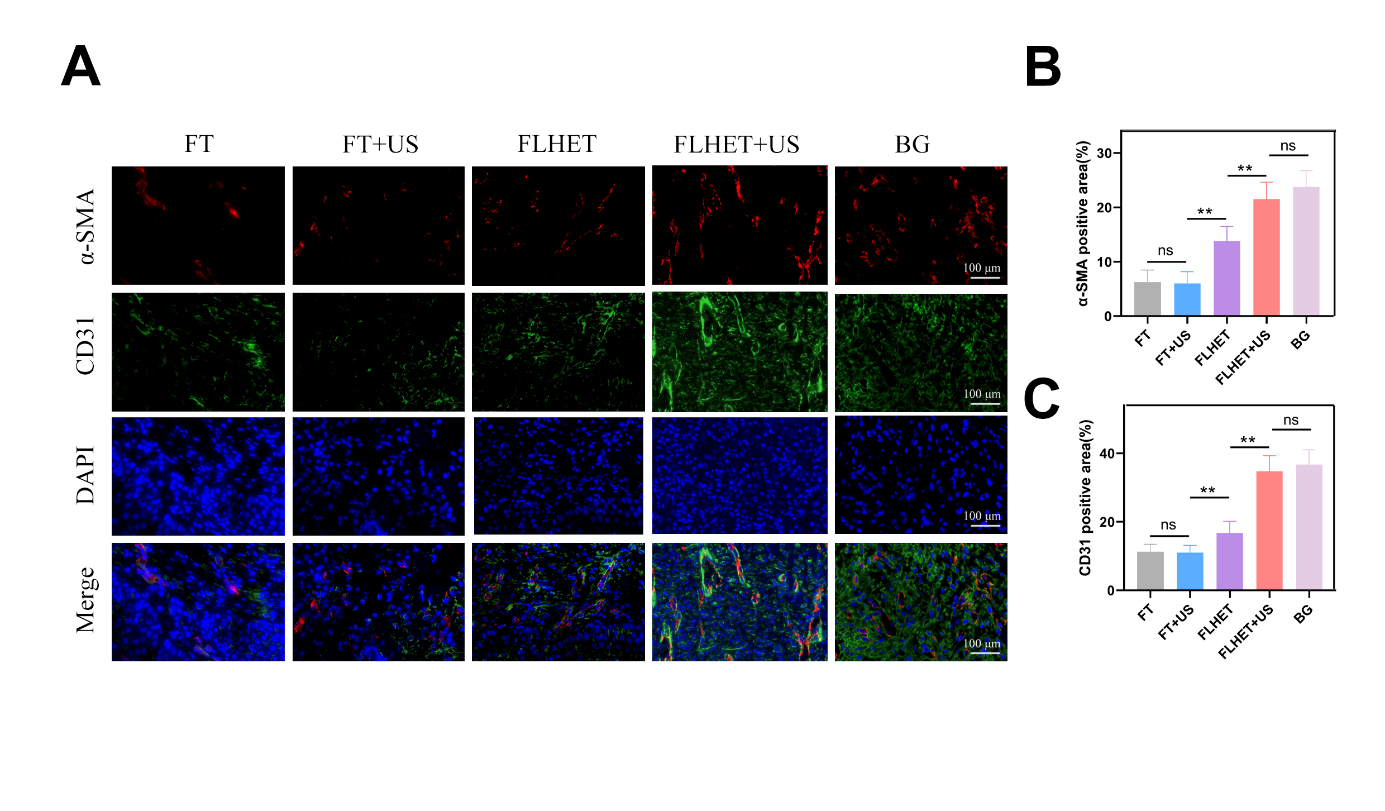


**Figure S15.** Representative immunofluorescence images (A) and quantification of CD31(B) and α-SMA (C)-positive areas in the tissues was performed on day 7 after the different treatments. Values were indicated as the mean±SD (n=4). Significance between two groups was calculated using one-way ANOVA and Tukey-Kramer multiple comparisons test. ns=no significance, **P* < 0.05, ***P* < 0.01.


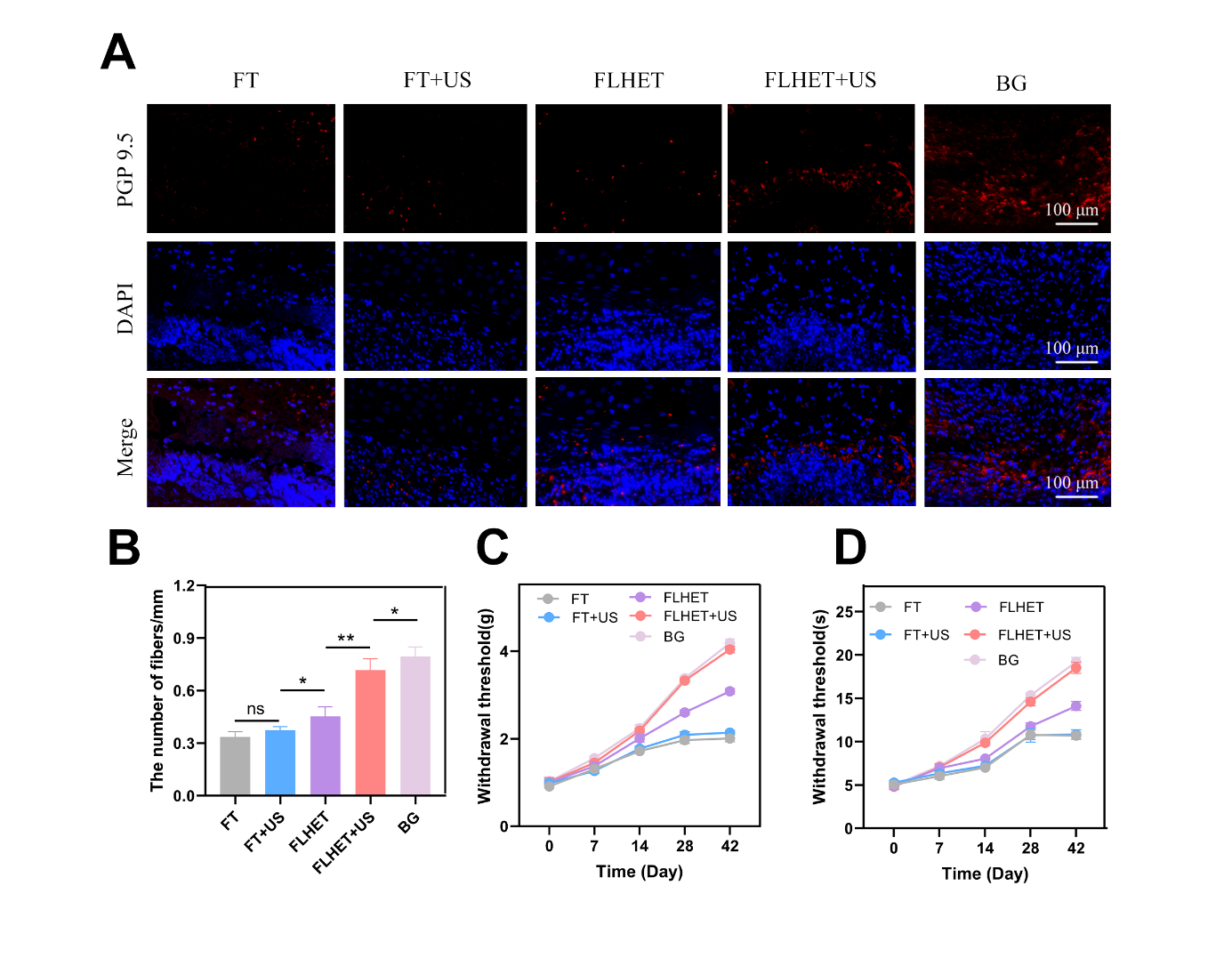


**Figure. S16.** *In situ* nanocomposite ‘lever’ hydrogels restored intraepidermal nerve fibre density (A, B) and improved thermal (C) and mechanical (D) sensitivity and in DFU rats. Values were indicated as the mean±SD (n=4). Significance between two groups was calculated using one-way ANOVA and Tukey-Kramer multiple comparisons test. ns=no significance, **P* < 0.05, ***P* < 0.01.


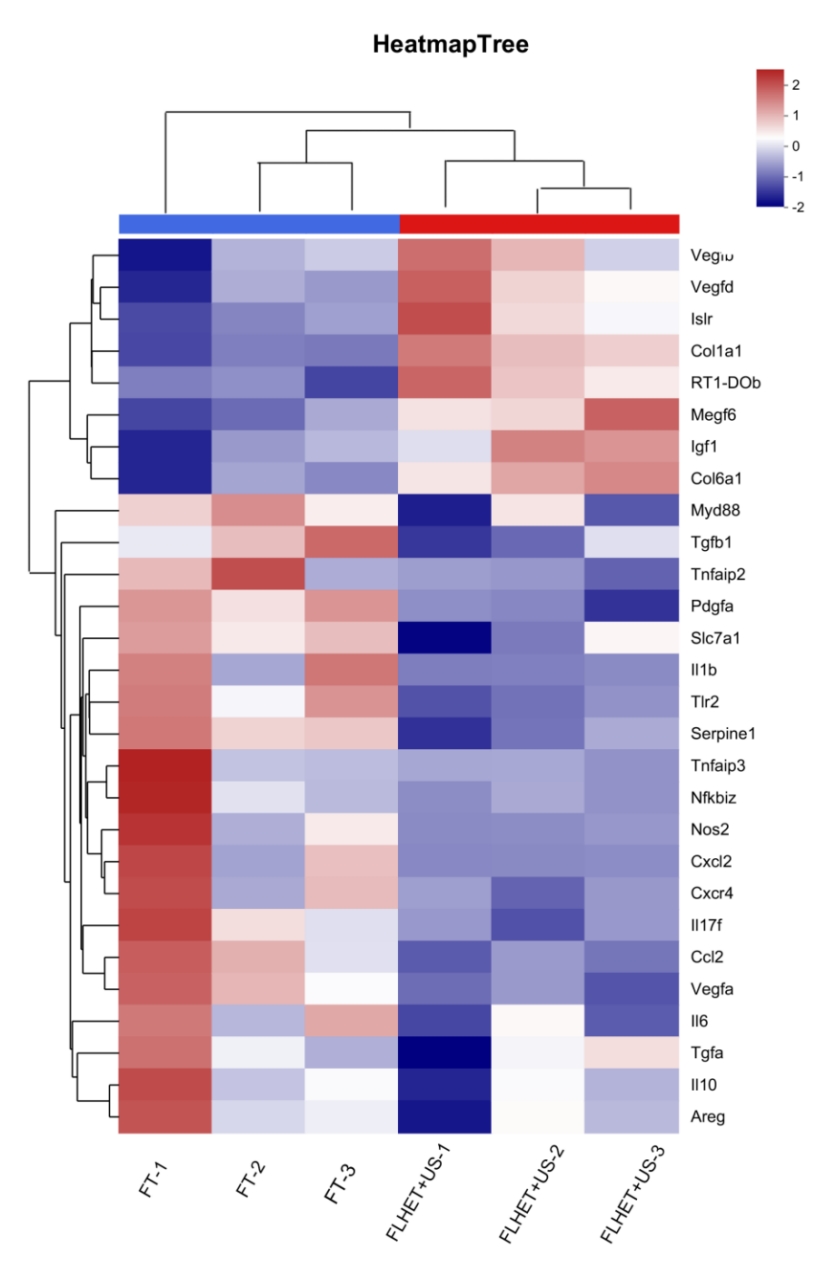


**Figure S17.** Heatmap changes in representative genes associated with inflammation between the control and FLHET+US-treated groups.


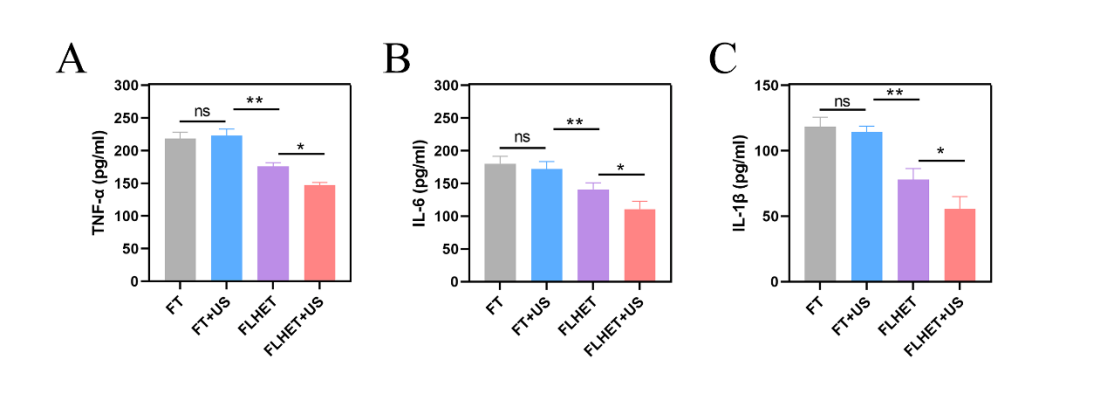


**Figure S18.** The level of TNF-α (A), IL-6 (B), and IL-1β (C) were determined via ELISA after different treatments. Values were indicated as the mean±SD (n=4). Significance between two groups was calculated using one-way ANOVA and Tukey-Kramer multiple comparisons test. ns=no significance, **P* < 0.05, ***P* < 0.01.


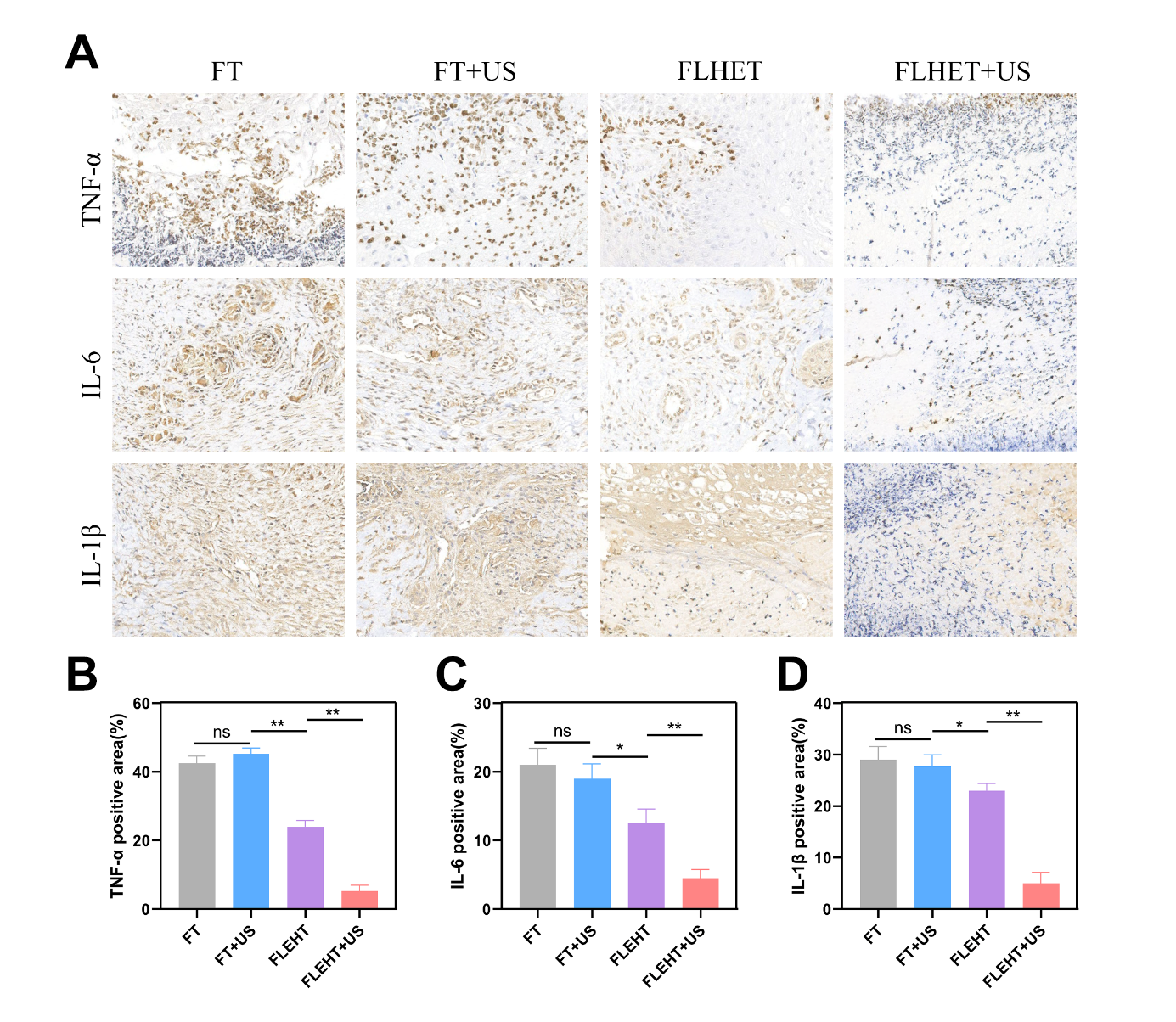


**Figure S19.** (A)The level of TNF-α, IL-6, and IL-1β were determined by immunohistochemical staining after different treatments. (B-D) Quantitative immunohistochemical analysis of TNF-α (B), IL-6 (C) and IL-1β (D) after different hydrogel treatments. Values were indicated as mean±SD (n=4). Significance between two groups was calculated using one-way ANOVA and Tukey-Kramer multiple comparisons test. ns=no significance, **P* < 0.05, ***P* < 0.01.

**Reference**

[1] Z. Lin, L. Y. Li, L. Chen, C. Jin, Y. Li, L. Yang, C. Z. Li, C. Y. Qi, Y. Y. Gan, J. R. Zhang, P. Wang, L. B. Ni, G. F. Wang, Acta Pharmacol Sin **2023**.

[2] J. Lou, H. Zhang, J. Qi, Y. Xu, X. Wang, J. Jiang, X. Hu, L. Ni, Y. Cai, X. Wang, W. Gao, J. Xiao, K. Zhou, Br J Pharmacol **2022**, 179, 301.

[3] Y. Qian, Y. Zheng, J. Jin, X. Wu, K. Xu, M. Dai, Q. Niu, H. Zheng, X. He, J. Shen, Adv Mater **2022**, 34, e2200521.

[4] X. Zhong, G. Wei, B. Liu, C. Wang, J. Wang, Y. Lu, W. Cui, H. Guo, Small **2023**, 19, e2207248.

[5] A. Enumo, Jr., D. F. Argenta, G. C. Bazzo, T. Caon, H. K. Stulzer, A. L. Parize, Int J Biol Macromol **2020**, 163, 167.

[6] Z. Zhou, T. Deng, M. Tao, L. Lin, L. Sun, X. Song, D. Gao, J. Li, Z. Wang, X. Wang, J. Li, Z. Jiang, L. Luo, L. Yang, M. Wu, Biomaterials **2023**, 299, 122141.

[7] D. Kim, G. Pertea, C. Trapnell, H. Pimentel, R. Kelley, S. L. Salzberg, Genome Biol **2013**, 14, R36.

[8] M. D. Robinson, D. J. McCarthy, G. K. Smyth, Bioinformatics **2010**, 26, 139.

[9] C. Xie, X. Mao, J. Huang, Y. Ding, J. Wu, S. Dong, L. Kong, G. Gao, C. Y. Li, L. Wei, Nucleic Acids Res **2011**, 39, W316.
